# Persistence of spike-specific immune responses in BNT162b2-vaccinated donors and generation of rapid *ex-vivo* T cells expansion protocol for adoptive immunotherapy: A pilot study

**DOI:** 10.3389/fimmu.2023.1061255

**Published:** 2023-02-02

**Authors:** Sarra Mestiri, Maysaloun Merhi, Varghese P. Inchakalody, Nassiba Taib, Maria K. Smatti, Fareed Ahmad, Afsheen Raza, Fatma H. Ali, Shereena Hydrose, Queenie Fernandes, Abdul W. Ansari, Fairooz Sahir, Lobna Al-Zaidan, Munir Jalis, Mokhtar Ghoul, Niloofar Allahverdi, Mohammed U. Al Homsi, Shahab Uddin, Andrew Martin Jeremijenko, Mai Nimir, Laith J. Abu-Raddad, Fatma Ben Abid, Ahmed Zaqout, Sameer R. Alfheid, Hassan Mohamed Hassan Saqr, Ali S. Omrani, Ali Ait Hssain, Muna Al Maslamani, Hadi M. Yassine, Said Dermime

**Affiliations:** ^1^ Translational Cancer Research Facility, National Center for Cancer Care and Research/ Translational Research Institute, Hamad Medical Corporation, Doha, Qatar; ^2^ National Center for Cancer Care and Research, Hamad Medical Corporation, Doha, Qatar; ^3^ Qatar University Biomedical Research Center, Qatar University, Doha, Qatar; ^4^ Translational Research Institute, Academic Health System, Hamad Medical Corporation, Doha, Qatar; ^5^ Dermatology Institute, Academic Health System, Hamad Medical Corporation, Doha, Qatar; ^6^ College of Medicine, Qatar University, Doha, Qatar; ^7^ Communicable Disease Center, Hamad Medical Corporation, Doha, Qatar; ^8^ Infectious Disease Epidemiology Group, Weill Cornell Medicine–Qatar, Cornell University, Qatar Foundation–Education City, Doha, Qatar; ^9^ World Health Organization Collaborating Centre for Disease Epidemiology Analytics on HIV/AIDS, Sexually Transmitted Infections, and Viral Hepatitis, Weill Cornell Medicine–Qatar, Cornell University, Qatar Foundation–Education City, Doha, Qatar; ^10^ Department of Population Health Sciences, Weill Cornell Medicine, Cornell University, New York, NY, United States; ^11^ Staff Medical Center, Department of Medicine, Hamad Medical Corporation, Doha, Qatar; ^12^ Medical Intensive Care Unit, Hamad Medical Corporation, Doha, Qatar

**Keywords:** SARS-CoV-2, COVID-19 vaccine, spike-specific immune responses, surrogate neutralization, spike-specific T cells expansion

## Abstract

**Introduction:**

The BNT162b2 mRNA-based vaccine has shown high efficacy in preventing COVID-19 infection but there are limited data on the types and persistence of the humoral and T cell responses to such a vaccine.

**Methods:**

Here, we dissect the vaccine-induced humoral and cellular responses in a cohort of six healthy recipients of two doses of this vaccine.

**Results and discussion:**

Overall, there was heterogeneity in the spike-specific humoral and cellular responses among vaccinated individuals. Interestingly, we demonstrated that anti-spike antibody levels detected by a novel simple automated assay (Jess) were strongly correlated (r=0.863, P<0.0001) with neutralizing activity; thus, providing a potential surrogate for neutralizing cell-based assays. The spike-specific T cell response was measured with a newly modified T-spot assay in which the high-homology peptide-sequences cross-reactive with other coronaviruses were removed. This response was induced in 4/6 participants after the first dose, and all six participants after the second dose, and remained detectable in 4/6 participants five months post-vaccination. We have also shown for the first time, that BNT162b2 vaccine enhanced T cell responses also against known human common viruses. In addition, we demonstrated the efficacy of a rapid ex-vivo T cell expansion protocol for spike-specific T cell expansion to be potentially used for adoptive-cell therapy in severe COVID-19, immunocompromised individuals, and other high-risk groups. There was a 9 to 13.7-fold increase in the number of expanded T cells with a significant increase of anti-spike specific response showing higher frequencies of both activation and cytotoxic markers. Interestingly, effector memory T cells were dominant in all four participants’ CD8+ expanded memory T cells; CD4+ T cells were dominated by effector memory in 2/4 participants and by central memory in the remaining two participants. Moreover, we found that high frequencies of CD4+ terminally differentiated memory T cells were associated with a greater reduction of spike-specific activated CD4+ T cells. Finally, we showed that participants who had a CD4+ central memory T cell dominance expressed a high CD69 activation marker in the CD4+ activated T cells.

## Introduction

In order to limit the rapid spread of severe acute respiratory syndrome coronavirus-2 (SARS-CoV-2), the causative agent of coronavirus disease 2019 (COVID-19), and its consequences across the globe, many efforts have been focused on developing safe and effective prophylactic vaccines (1). The BNT162b2 vaccine (Pfizer-BioNTech) was the first vaccine to be authorized for emergency use (2). BNT162b2 is a lipid nanoparticle formulated nucleoside-modified messenger RNA (mRNA) encoding the SARS-CoV-2 full-length spike (S) glycoprotein in a prefusion stabilized conformation (2). The vaccine was found safe and demonstrated 95% efficacy for protection against COVID-19 in phase II/III clinical trials (3). Observational data showed that BNT162b2 is highly effective in preventing SARS-CoV-2 infection, related hospitalization, and death (4). However, limited data exist about the persistence of the humoral and T cell responses and the duration of the vaccine-induced protection after the two-dose mRNA vaccination.

Neutralizing antibodies are the best indicators of protective immunity, therefore the quantification of SARS-CoV-2 neutralizing antibody levels induced by vaccination or infection constitutes a critical parameter to determine the protection level against the virus and to assess the potential vaccine effectiveness (5–7). The conventional virus/pseudovirus neutralization assays are considered the reference methods to determine the functional neutralizing ability of antibodies (8, 9). However, these methods require the use of specialized facilities, trained personnel, are time-consuming (2-4 days), and relatively expensive (8, 9). Several surrogates of neutralization cell-based assays have been developed and evaluated to overcome these limitations (10–13). Most of these assays use ELISA or similar platforms requiring multiple time-consuming binding and washing steps (10–13), thus preventing high-throughput screening. Therefore, simple, rapid, and accurate serological tests measuring neutralizing activity are urgently needed to assess the duration of humoral protective immunity in vaccine recipients and in recovered COVID-19 patients. Various new techniques have been employed globally for antibody response monitoring following SARS-CoV-2 infection or immunization (14). Jess Simple Western system is a novel fully automated assay, from Protein Simple, that detects human serum/plasma binding antibodies reactive to five different SARS-CoV-2 viral antigens in a large number of samples in only three hours (15). At the beginning of the pandemic, this assay showed high utility in COVID-19 diagnosis with a sensitivity and specificity of 94% and 93%, respectively (16). Moreover, Jess revealed a substantial agreement of 90% between the results obtained using Jess and ELISA for SARS-CoV-2 Immunoglobulin G (IgG) detection, which substantiates its implementation as a first-line serological test for clinical diagnostics and vaccination monitoring (16). Subsequently, Jess was then used in several studies to characterize SARS-CoV-2 specific humoral response in animal and human systems (17–19).

Alongside the antibody response, recent studies have shown that T cell response plays a dominant role in SARS-CoV-2 viral clearance and protection (20–24). Indeed, several reports indicated that COVID-19 patients with undetectable or impaired humoral responses could recover from the disease, highlighting the importance of the T cell response in virus clearance (20–22). In addition, Hurme et al. demonstrated that T cell memory response in COVID-19 vaccinated, and convalescent individuals could be more persistent than antibody response leading to a more durable source of protection (23, 24). Furthermore, recent studies indicated that T cell response and functionality against SARS-CoV-2 were not affected by the mutations or antigenic variation of the emerging variants of concern as the humoral response (25–27). These findings provide direct evidence that a lack or impairment of the T cell response could be associated with an elevated risk of SARS-CoV-2 infection and severe COVID-19 disease outcome. In the same line, numerous studies indicated that severe outcome in COVID-19 patients was associated with lymphopenia, reduction or disability of the T cell cytotoxic potential, and elevated exhaustion markers (28–30). On the other hand, recent studies demonstrated that immunocompromised patients and the elderly have poor immune responses to the BNT162b2 vaccine, indicating that these patients may not be sufficiently protected against SARS-CoV-2 infection (31–33). Therefore, the development of new therapies that support the cellular response to SARS-CoV-2 by preventing the defect of T cell function may have a significant impact on the outcome of the elderly, immunocompromised, and severe COVID-19 patients after infection or vaccination.

We herein report the dynamics and persistence of antibody and T cell responses in a small cohort of healthy adult recipients of two doses of BNT162b2-mRNA vaccine in the state of Qatar. In addition, we explored the interpersonal variation of the humoral and cellular immune response elicited by BNT162b2 immunization among vaccinated healthy individuals. We further evaluated the Jess technology as a surrogate assay for SARS-CoV-2 antibodies neutralizing activity estimation. Finally, we explored the feasibility and efficacy of a rapid *ex-vivo* T cell expansion protocol for spike-specific T cell expansion to be potentially used for adoptive-cell therapy in severe COVID-19, immunocompromised patients, and elderly persons.

## Material and methods

### Study population and sample collection

This study was conducted at the Translational Cancer Facility, National Center for Cancer Care and Research, Hamad Medical Corporation (HMC), Qatar. A total of six healthy participants with no history of SARS-CoV-2 infection, eligible to receive two doses of the BNT162b2 mRNA vaccine (Pfizer-BioNTech) three weeks apart, were enrolled. For each participant, peripheral blood samples were obtained on day 0 (prior vaccination), day 20 (pre-boost), day 34 (14 days post-boost), and day 150 (five months after the first vaccination dose). Peripheral Blood Mononuclear Cells (PBMCs) and serum were isolated and used for serological and T cell responses analysis as reported in **supplement 1** (See **supplementary material**). Demographic characteristics of enrolled participants have been shown in **supplement 2** (See **supplementary material**). This study was approved by the Institutional Review Board (IRB) committee of HMC (Project number MRC-01-21-113), and informed consent was obtained from all study participants.

### PBMCs and sera isolation

Peripheral blood samples were collected in EDTA and serum separator tubes at the different time points reported above. Serum was separated by centrifugation at 3200 rpm and stored at -80°C. PBMCs were isolated by density gradient centrifugation using Ficoll Paque Premium (GE Healthcare) and SepMate tubes (STEMCELL Technology) according to the manufacturer’s instructions. Isolated PBMCs were then cryopreserved in a cell recovery medium (Fetal Bovine Serum (FBS, Gibco) supplemented with 10% DMSO (Millipore Sigma) and stored in vapor phase liquid nitrogen until used.

### ELISA binding assay

Initially, sera samples were screened for the presence of IgG antibodies against the SARS-CoV-2 recombinant S1 subunit (S1) of the spike protein, using a commercial semi-quantitative ELISA kit (Lionex COVID-19 ELISA-human IgG) as per the manufacturer’s instructions. Briefly, sera samples were diluted at 1:50 in a sample diluent and then added to the microtiter plate (coated with SARS-CoV-2 S1 protein) for 60 mins incubation at room temperature. After a washing step, the conjugate (peroxidase-coupled anti-human antibody) and its substrate (TMB) were added to the wells. The optical density (OD) was measured with an ELISA reader (Epoch Biotek) at 450 nm wavelength. Each sample OD was normalized according to the kit calibrator value, and this normalized value determines the test result. Values below 0.8 were considered negative, values between 0.8 and 1.1 were considered borderline, and values above 1.1 indicate a positive anti-SARS-CoV-2 S1 subunit IgG. All samples were run in duplicates and borderline samples were repeated for confirmation.

### Detection of SARS-CoV-2 specific antibodies using jess simple western system

The detection and quantification of anti-SARS-CoV-2 IgG antibodies among vaccinated donors’ sera were assessed using the Jess Simple Western system (Protein Simple). This system enables the detection of human IgG antibodies reactive against five viral antigens simultaneously: S1 Receptor Binding Domain protein (S1-RBD), S1 subunit full length (S1), S2 subunit full length (S2), Spike protein (S), and Nucleocapsid Protein (N) recombinant antigens as reported in **supplement 3** (see **supplementary material**). Samples were run following the manufacturer’s protocol for the 12-230-kDa Jess separation module (Protein Simple). Briefly, the SARS-CoV-2 antigens (Protein Simple) were mixed with 0.1X Sample buffer (Protein Simple) and Fluorescent 5X Master mix (Protein Simple) in the presence of fluorescent molecular weight markers (Protein Simple) and denatured at 95°C for 5 mins. Sera were diluted at 1:10 in the sample buffer. Ladder (12-230-kDa PS-ST02EZ, Protein Simple) and SARS-CoV-2 proteins were run in capillaries. The SARS-CoV-2 specific human antibodies present in the serum samples serve as primary antibodies that were then detected with anti-goat HRP-conjugated anti-human IgG antibody (R&D Systems). The chemiluminescent revelation was established with peroxide/luminol-S (Protein Simple). The digital image of the capillary chemiluminescence was captured with Compass Simple Western software (version 4.1.0, Protein Simple) that automatically calculated the area of the signal (chemiluminescence intensity). Results are represented as the chemiluminescence intensity of each antigen separately.

### Generation of SARS-CoV-2 pseudotyped vesicular stomatitis virus and neutralization assay

For the determination of neutralizing antibodies to SARS-CoV-2, we utilized a recombinant ΔG-Vesicular stomatitis virus (VSV) system to generate SARS-CoV-2 pseudovirus as previously described by Whitt (34). Briefly, HEK293T cells were grown in DMEM medium (Gibco) supplemented with 10% Fetal Bovine Serum (Gibco) and 1% Pen/Strep (Gibco) to reach 80-90% of confluence on the day of the experiment. The following day, cell culture media was replaced with Opti-MEM (Gibco) and incubated for 20 mins before transfecting cells with SARS-CoV-2 Spike-TM plasmid (provided by the Viral Pathogenesis Laboratory, Vaccine Research Center, National Institute of Health). After 4 hours, transfection media was replaced with DMEM (Gibco) supplemented with 5% FBS (Gibco), and cells were incubated at 37°C with 5% CO_2_. After 24 hours, cells were examined for the presence of syncytia due to the expression of the envelope protein. Subsequently, transfected cells were infected with pseudotyped ΔG-luciferase (G*ΔG) (Kerafast, Ref. no. EH1025-PM) at a multiplicity of ~3–5. When most of the cells showed a cytopathic effect (24-30 hours), SARS-CoV-2 VSV pseudovirus was harvested by collecting the supernatant. Supernatants were clarified by centrifugation at 300×g for 10 mins before aliquoting and storing at −80°C. For the titration of pseudotyped viruses, HEK293T cells expressing angiotensin-converting enzyme 2 (ACE2) (BEI) were used. Cells were prepared at 1×10^6^ cell/ml in complete DMEM (Gibco) and added to serially diluted pseudovirus (50 µl of diluted virus added to 50 µl of cells in suspension) in a 96-well cell culture plate and incubated for 2 hours. 100 µl of complete DMEM (Gibco) was then added to the cells and incubated for 48 hours. After incubation, cells were lysed using 30 µl of 1X cell lysis buffer (Promega), and 50 µl of luciferase reagent (Promega) was added. The titer of the pseudovirus was determined by measuring luminescence using a plate reader (Tecan Infinite). To assess the neutralization of SARS-CoV-2 pseudotyped VSV in sera samples, heat-inactivated serum samples (50 to 200-fold) were serially diluted in 60 µl of DMEM media and then incubated with 100 µl pseudovirus (titer 1-2×10^6^ RLU/100 µl) for 30 mins at room temperature. The final volume (160 µl) was then distributed into 3 wells (triplicates) of a 96-cell culture plate. HEK293T-ACE2 cells were then added at 1×10^6^ cells/ml and incubated for 48 hours before reading out luminescence using a plate reader (Infinite 200 PRO). A positive response was defined as a neutralizing activity of 20% or more.

### Interferon-γ Enzyme-Linked ImmunoSpot assay

The spike-specific T cell responses to the BNT162b2 vaccine were assessed using the T-spot Discovery SARS-CoV-2 kit (Oxford Immunotec), a modified enzyme-linked immunospot technology. This kit is designed to measure interferon-γ responses to overlapping peptide pools covering peptide sequences of five different SARS-CoV-2 antigens, without HLA restriction. The test specificity to SARS-CoV-2 has been enhanced by removing high homology peptide sequences that are potentially cross-reactive with other coronaviruses. The T-spot discovery SARS-CoV-2 kit was used according to the manufacturer’s protocols. Briefly, 250 000 PBMCs suspended in AIM-V medium (Gibco) were plated into each well of the T-spot plate in duplicates, stimulated with 3 different antigens: S1 spike subunit peptides, peptides coding for sequences with high homology to other coronaviruses, positive control (phytohemagglutinin), and negative control (AIM-V medium) then incubated for 18 hours (37°C, 5% CO_2_). The interferon-γ secreting T cells were detected using an automated ELISpot reader (Autoimmun Diagnositka GMBH). Results are presented as the mean of the number of spots forming cells (SFC) per 250 000 cells for each panel, subtracting the background (negative control) count. A positive response was defined as an SFC of 10 or more.

### *Ex-vivo* spike-specific T cells expansion

The spike-specific T cells were expanded from vaccinated donors’ PBMCs (collected five months post-vaccination) using a modified protocol for expansion of multivirus-specific T cells targeting cytomegalovirus (CMV), Epstein-Barr virus (EBV), BK virus (BKV), human herpes virus (HHV)-6, respiratory syncytial virus (RSV), adenovirus (Adv) and influenza previously described by Gerdemann et al. (35). Briefly, fresh PBMCs were pulsed with the spike peptide pools at 1 µg of antigen/15 x 10^6^ PBMCs for 30 mins at 37°C. The spike peptide pools (JPT Peptide Technologies) contain a pool of 315 overlapping peptides encompassing the full spike protein. After incubation, cells were resuspended in a virus-specific T cells (VST) medium consisting of 45% Advanced RPMI 1640 (Gibco) supplemented with 45% Click’s medium (Irvine Scientific), 2 mM GlutaMAX (Gibco), 10% FBS (Gibco), 10 ng/ml interleukin 7 (IL-7, Peprotech), and 400 U/ml IL-4 (Peprotech) and transferred to a G-Rex 10 device (Wilson Wolf Manufacturing Corporation). Cells were counted on day six and fresh culture media with cytokines was added. Cells were harvested and evaluated for antigen specificity and functionality on day 11.

### Flow cytometry

Expanded spike-specific T cells and PBMCs collected five months post-vaccination were stimulated with the S1 peptide pools (1µg/ml, Oxford Immunotec) for 18 hours. Stimulated PBMCs and T cells were stained with fluorophore-conjugated monoclonal antibodies against CD3 (BD Biosciences), CD4 (BD Biosciences), CD8 (BD Biosciences), CD45RA (BD, Biosciences), CD69 (BD Biosciences), CD107 (BD Biosciences), CD134 (Thermo Fisher), CD137 (Thermo Fisher), and CD197 (BD Biosciences) for phenotypical characterization. All samples were acquired using a Fortessa flow cytometer (BD Biosciences) and the data was analyzed using FlowJo V10 software (BD Biosciences).

### Statistical analyses

All statistical analyses and graphs were performed using GraphPad Prism Software (version 9.2.0). The characterization of the humoral and T cell responses dynamics over time was assessed using One-way ANOVA multiple comparison test. The T cell response to S1 antigen before and after expansion was evaluated using the student t test. Correlations between Jess, neutralization, and ELISA immunoassays were analyzed by Pearson correlation and linear regression models. The scatter point represents serum samples (n=24) collected from BNT162b2 vaccinated healthy donors at the baseline, 20-, 34-, and 150-days post-vaccination. The coefficient of correlation (r) represents the strength of the linear relationship between the different immunoassays. The coefficient of determination R squared (R2) represents the percentage of variance in the given data set. The P-value tests whether the regression equation is significant. P-value was considered statistically significant when P ≤ 0.5.

## Results

### Heterogeneity of the spike-specific antibody response among BNT162b2 vaccinated individuals

**Figure 1:**
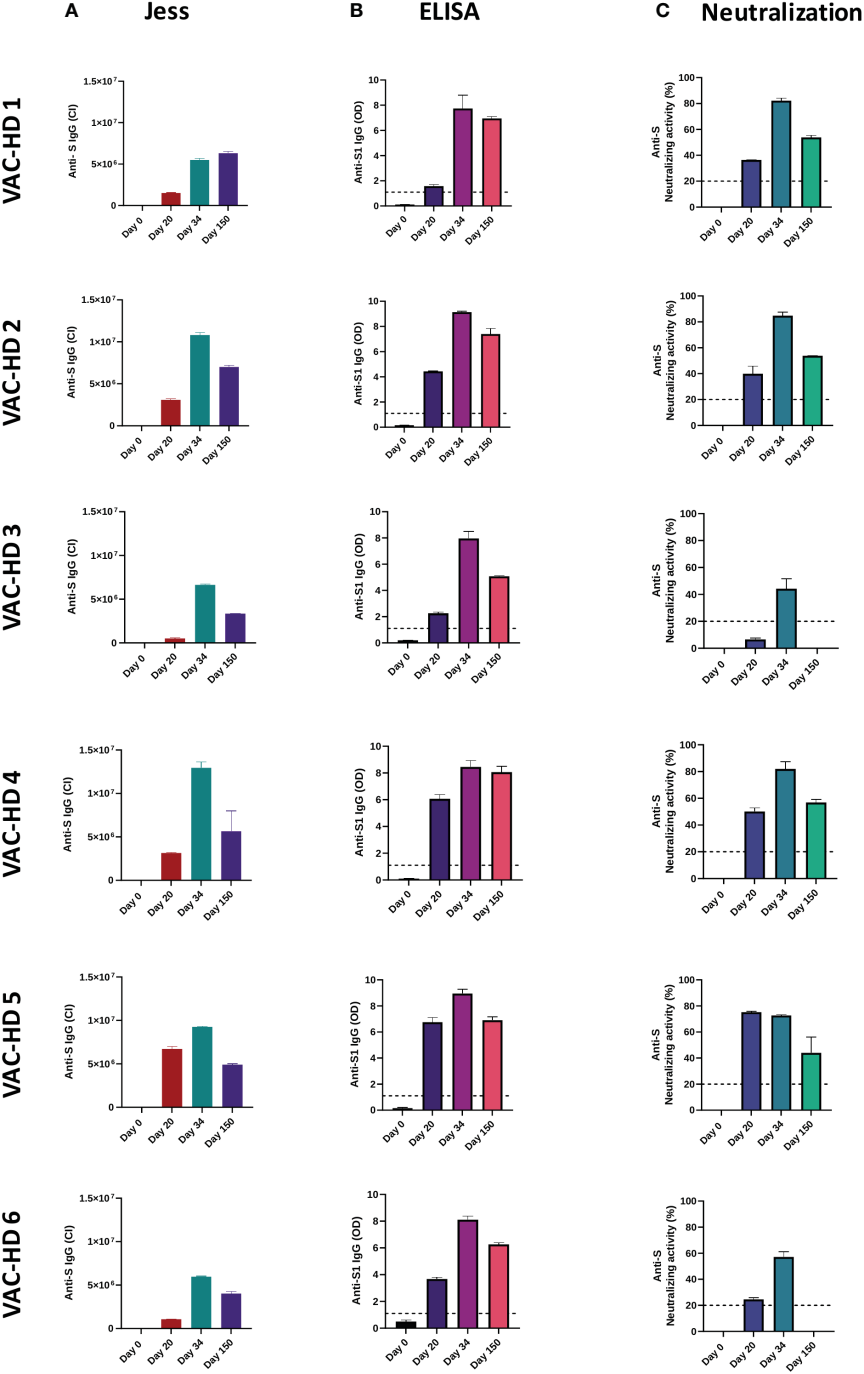
Interpersonal variation of anti-spike binding and neutralizing antibodies responses over time among BNT162b2 vaccinated participants. Serum samples were collected from six BNT162b2 vaccinated participants at the baseline, 20-, 34-, and 150-days post-vaccination. The anti-S and anti-S1 IgG levels were assessed using Jess and ELISA, respectively. The anti-S neutralizing activity was measured using the neutralization assay. **(A)** Anti-S IgG response in six BNT162b2 vaccinated participant over time (Jess). **(B)** Anti-S1 IgG response in six BNT162b2 vaccinated participant over time (ELISA). **(C)** Anti-S neutralizing activity in each BNT162b2 vaccinated participant over time (Neutralization assay). The lines indicate the cut-off value of a positive antibody response.

**Figure 2:**
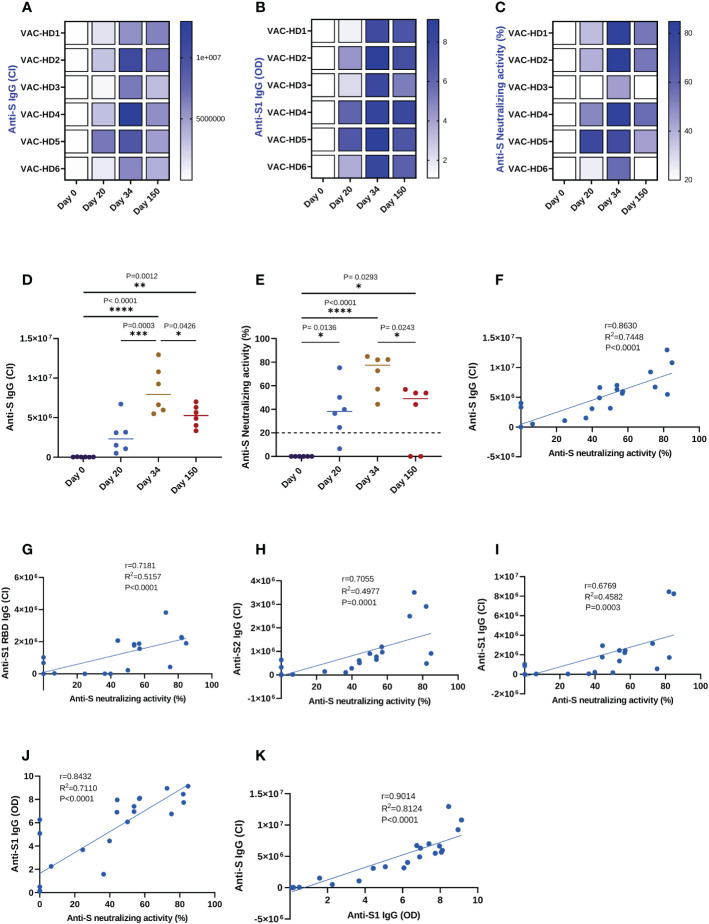
Correlation between the anti-spike binding and neutralizing antibodies responses induced by BNT162b2 vaccination and detected by three different immunoassays. Serum samples were collected from six BNT162b2 vaccinated participants at the baseline, 20-, 34-, and 150-days post-vaccination. The anti-S and anti-S1 IgG levels were assessed using Jess and ELISA, respectively. The anti-S neutralizing activity was measured using the neutralization assay. **(A–C)** Heat-map of anti-S IgG, anti-S1 IgG, and anti-S neutralizing activity responses in six BNT162b2 vaccinated participants over time. **(D)** Dynamics of the anti-S IgG levels in six BNT162b2 vaccinated participants over time (Jess). **(E)** Dynamics of the anti-S neutralizing activity in six BNT162b2 vaccinated participants (Neutralization assay). Each symbol represents an individual participant with a line indicating the median of each time point. One-way ANOVA test was used, P value was considered statistically significant when *P ≤ 0.05. All samples were run in duplicates. **(F–I)** Correlation between anti-S, S1 RBD, S2, and S1 IgG levels detected by JESS and neutralizing activity, respectively. **(J)** Correlation between anti-S1 IgG levels detected by semi-quantitative ELISA and neutralizing activity. **(K)** Correlation between anti-S1 IgG levels detected by ELISA and anti-S IgG levels detected by JESS. All correlations were analyzed by Pearson statistical test and linear regression models. The scatter point represents serum samples (n=24) collected from six BNT162b2 vaccinated participants at the baseline, 20-, 34-, and 150-days post-vaccination, and the blue error band represents the 95% confidence interval. The coefficient of correlation (r) represents the strength of the linear relationship between the different immunoassays. The coefficient of determination (R2) represents the percentage of variance in the given data set. The P-value tests whether the regression equation is significant. P value was considered statistically significant when *P ≤ 0.05. The stars present the level of significance. *P < 0.05; **P < 0.01; ***P < 0.001; ****P < 0.0001.

The anti-spike (anti-S) binding and neutralizing antibody responses induced by the BNT162b2 vaccine over time were characterized. In this, serum samples were collected from vaccinated participants at four different time points as reported in **supplement 1** (See **supplementary material**). The anti-S and anti-S1 IgG levels were assessed using Jess and ELISA, respectively (**Figures 1A, B**, **2A, B** and **supplement 4**). The anti-S neutralizing activity was measured using the neutralization assay (**Figures 1C** and **2C**). Overall, our data showed an interpersonal heterogeneity in the vaccine-elicited humoral response among vaccinated individuals. This interpersonal variation was observed at three stages: the induction detected on day 20, the peak response reached on day 34, and the response decline detected 150 days post-vaccination (**Figures 1A–C**, **2A–C**). Jess results showed that the anti-S IgG antibody response (presented by chemiluminescence intensity (CI)) was induced on day 20 in all six participants with different levels, ranging from 502947 to 6719958 CI (**Figures 1A** and **2A**). This response was boosted on day 34 (after the second dose) in all six participants with varying levels, ranging from 5495488 to 12954728 CI (**Figures 1A** and **2A**). However, 150 days post-vaccination a decline in the anti-S IgG levels was observed in 5/6 participants with different magnitudes ranging from 3344080 to 7009985 CI (**Figures 1A** and **2A**). In contrary, VAC-HD1 showed an increase in this response 150 days post-vaccination (**Figures 1A** and **2A**).

In addition, ELISA results showed that the first vaccination induced the anti-S1 IgG antibody response on day 20 in all six participants with varying degrees of optical density (OD) ranging from 1.58 to 6.75 (**Figures 1B** and **2B**). The second dose increased this response in all six participants with an OD ranging from 7.73 to 9.13 (**Figures 1B** and **2B**). However, five months post-vaccination a decline in the anti-S1 IgG levels was observed in all six participants with an OD ranging from 5.07 to 8.06 (**Figures 1B** and **2B**). We have found also that females (VAC-HD2, 4, 5, and 6) had a stronger anti-S1 IgG antibody response on days 20 and 34 compared to their counterparts in males (VAC-HD1 and 3) (**Figures 1B** and **2B**).

Furthermore, Neutralization results showed that anti-S neutralizing antibodies response was induced on day 20 in all six participants with different levels of neutralizing activity ranging from 6.53 to 75.23% (**Figures 1C** and **2C**). This response was boosted after the second dose in 5/6 participants with a neutralizing activity ranging from 44.27 to 84.76% (**Figures 1C** and **2C**). However, five months post-vaccination a decline in the anti-S neutralizing activity was observed in all six participants with varying magnitudes ranging from 0 to 56.84% (**Figures 1C** and **2C**). Interestingly, we demonstrated that individuals who had low Anti-S neutralizing activity (<25%) on day 20 (VAC-HD3 and 6) tended to have also low response after the second dose and lost this response five months post-vaccination (**Figures 1C** and **2C**). However, individuals who had high anti-S neutralizing activity (>36%) on day 20 (VAC-HD1, 2, 4, and 5) tended to have also high response after the second dose and were able to maintain this response five months post-vaccination (**Figures 1C** and **2C**).

### Dynamics of S-specific binding and neutralizing antibody responses following BNT162b2 vaccination

Our data showed that anti-S binding and neutralizing antibodies responses dynamics followed the same trend (**Figures 2D, E**). Indeed, the anti-S IgG binding antibodies (BAbs) response was induced 20 days after the first dose (26526 CI on day 0 versus 2678053 CI on day 20), significantly boosted with the second dose (2678053 CI on day 20 versus 8519960 CI on day 34, ***P=0.0003), then significantly declined 150 days post-vaccination (8519960 CI on day 34 versus 5205907 CI on day 150, *P=0.0426) (**Figure 2D**). Identically, we found that the anti-S neutralizing antibodies (NAbs) response was significantly induced 20 days after the priming dose (0% on day 0 versus 38.8% on day 20, *P=0.0136) and was further increased with the booster dose (38.8% on day 20 versus 70.51% on day 34) (**Figure 2E**). However, the anti-S neutralizing activity had significantly decreased five months post-vaccination as compared to their peak levels at two weeks after the second dose (70.51% on day 34 versus 34.77% on day 150, *P=0.0243) (**Figure 2E**). Furthermore, we demonstrated that all six participants maintained a detectable anti-S BAbs response five months post-vaccination (**Figures 2A** and **2D**), whereas only four of them maintained the NAbs response (except VAC-HD3 and VAC-HD6) (**Figures 2C, E**).

### Evaluation of Simple automated immunoassay Jess as an alternative to neutralization cell-based assay for SARS-CoV-2 neutralizing activity estimation

We investigated whether the anti-S IgG BAbs levels measured by Jess can substitute the neutralization cell-based assay for the estimation of neutralizing activity in vaccinated individuals. For this purpose, the anti-S BAbs levels and neutralizing activity of anti-S NAbs were measured in serum samples (n=24) collected at the baseline, 20-, 34-, and 150-days post-vaccination using Jess, ELISA, and neutralization assays in order to evaluate the degree of correlation between these 3 immunoassays. We first performed correlation and linear regression analysis on the four different BAbs (anti S, anti-S1 RBD, anti-S2, and anti-S1 IgG) levels detected by Jess and neutralizing activity measured by neutralization assay (**Figures 2F–I**). Among the four BAbs, anti-S IgG showed a strong positive, statistically significant correlation (r=0.8630, R^2 =^ 0.7448, P <0.0001) with the neutralizing activity (**Figure 2F**), whereas a moderate correlation (r ranging between 0.6769 and 0.7181) was observed for the remaining IgGs (anti-S1 RBD, anti-S1, and anti-S2) (**Figures 2G–I**). We then evaluated whether Jess is a better surrogate test for neutralizing activity prediction as compared to another common commercial semi-quantitative ELISA test. Linear regression analysis showed that the linear fit between anti-S IgG detected by Jess and neutralizing activity (R^2 =^ 0.7448, P<0.0001) was substantially higher as compared to the one obtained between anti-S1 IgG levels detected by ELISA and neutralizing activity (R^2 =^ 0.7110, P<0.0001) (**Figure 2J**). Similarly, a distinguished positive correlation (r=0.9014, R^2 =^ 0.8124, P<0.0001) between anti-S1 IgG and anti-S IgG levels detected by ELISA and Jess respectively was observed (**Figure 2K**). Overall, these results indicate that the detection of anti-S IgG levels by Jess could be a better surrogate for neutralizing activity estimation compared to ELISA. Moreover, Jess could potentially be a promising alternative that is quicker, cheaper, and easier than the conventional cell-based assays for neutralizing activity estimation.

### BNT162b2-induced T cells response to SARS-CoV-2 and cross-reactivity with other viral antigens

**Figure 3:**
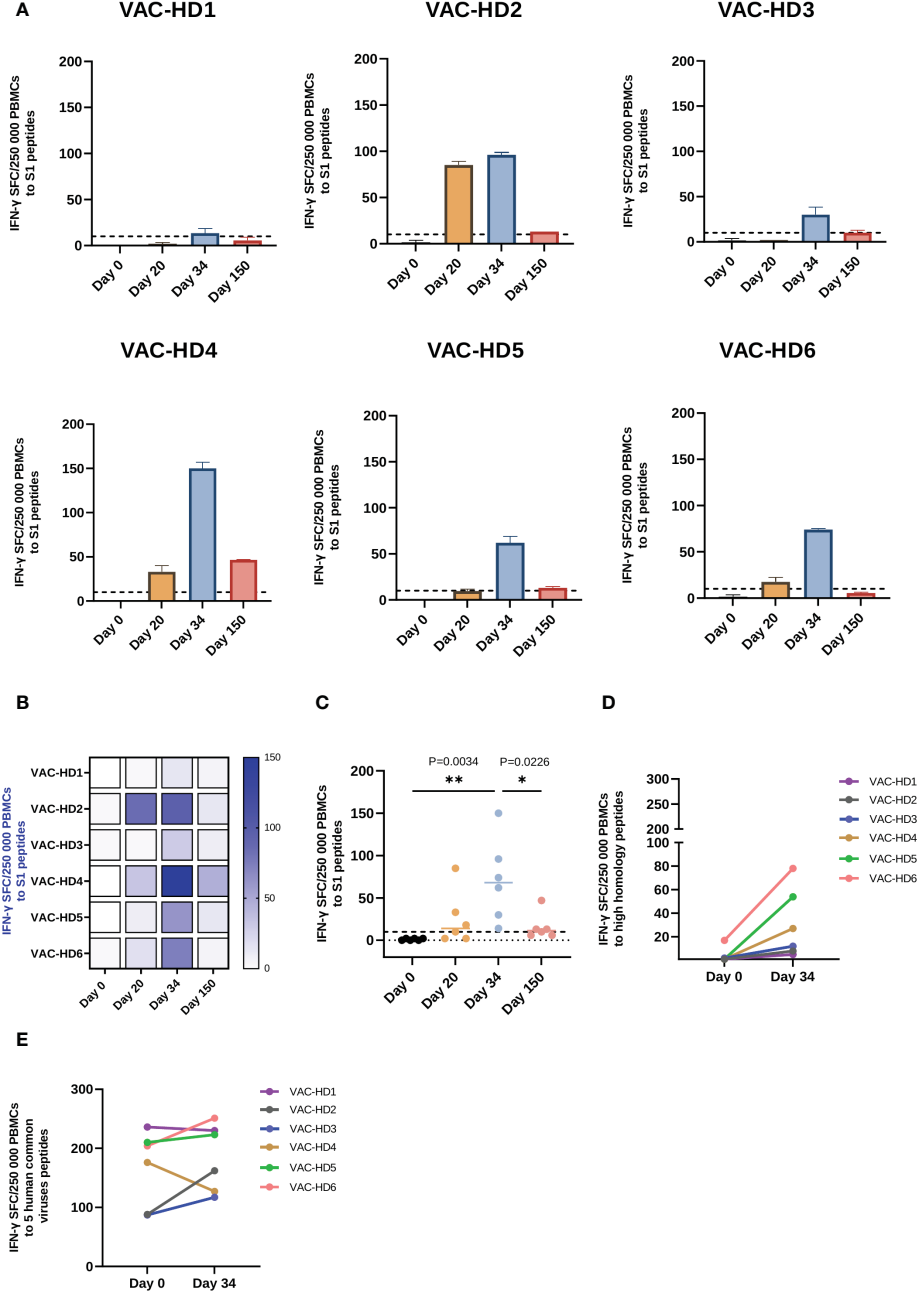
Spike-specific and spike cross-reactive T cell responses in BNT162b2 vaccinated participants. **(A)** T cell response to S1 peptide pools in six BNT162b2 vaccinated participant at the baseline, 20-, 34-, and 150-days post-vaccination. **(B)** Heat-map of S1- specific T cell responses in six BNT162b2 vaccinated participants over time **(C)** Dynamics of S1- specific T cell responses in six BNT162b2 vaccinated participants over time. Each symbol represents an individual participant with a line indicating the median of each time point. **(D)** T cell response to high homology peptide pools in BNT162b2 vaccinated participants at baseline (Day 0) and on day 34 post-vaccination (after the second dose) **(E)** T cell response to five human common viruses peptides (CMV, EBV, AdV 3 and 5 and BKV) in BNT162b2 vaccinated participants at baseline (Day 0) and on day 34 post-vaccination (after the second dose). Results are presented as the mean of the number of spots forming cells (SFCs) per 250 000 PBMCs subtracting the background (negative control) count. A positive response was defined as an SFCs of 10 or more. One-way ANOVA test was used, P value was considered statistically significant when *P ≤ 0.05. All samples were run in duplicates. The stars present the level of significance. *P < 0.05; **P < 0.01.

The cellular immune responses induced by BNT162b2 vaccination were characterized by the measurement of interferon-γ responses to S1 peptide pools (the immunodominant subunit of the S protein) using a relatively novel T-spot assay. This assay is highly specific since the SARS-CoV-2 epitopes having a high degree of homology with other endemic human coronaviruses (huCoVs) were removed from the SARS-CoV-2 antigens panels enabling a specific SARS-CoV-2 response determination. Similar to the antibody response, we observed an immense variation in the T cell responses among the vaccinated participants (**Figures 3A, B**). T-spot results showed that S1-specific T cell response was significantly induced on day 20 in 5/6 participants with variable levels ranging from 2 to 85 SFC (**Figures 3A, B**). An increase in this response was observed in all six participants on day 34 with an S1-specific T cell response ranging from 14 to 150 SFC (**Figures 3A, B**). However, 150 days post-vaccination a decline in the S1-specific response was observed in all six participants with different magnitudes ranging from 6 to 47 SFC (**Figures 3A, B**). We have found that females (VAC-HD 2, 4, 5, and 6) had a stronger S1-specific T cell response on days 20 and 34 compared to their counterparts in males (VAC-HD1 and 3) (**Figures 3A, B**).

On the other hand, we have demonstrated that the priming dose was able to induce a detectable T cell response (≥ 10 SFCs) against the S1 antigen in only 4/6 participants, whereas after the booster dose, all six participants presented a detectable T cell response ranging from 14 to 150 SFC (**Figure 3C**). Interestingly, we have shown that T cell response against the S1 antigen was induced 20 days after the priming dose (1 SFC on day 0 versus 25 SFCs on day 20) and significantly increased two weeks after the second dose (1 SFC on day 0 versus 71 SFC on day 34, **P=0.0034) in all the participants (**Figure 3C**). However, we have observed that T cell response to the S1 antigen was significantly decreased 150 days post-vaccination (71 SFC on day 34 versus 15.83 SFC on day 150, * P=0.0226) (**Figure 3C**). This decline was observed in all six participants, whereas this T cell response remained detectable 150 days after vaccination in 4/6 participants (**Figures 3B, C**).

Given the fact that SARS-CoV-2 displays a high level of homology to other human coronaviruses (huCoVs), we evaluated whether the BNT162b2 vaccination could induce a cellular immune response against other huCoVs strains than SARS-CoV-2. Therefore, we compared the T cell response, using high homology peptide pools, on day 0 and day 34 post-vaccination. Interestingly, all six participants showed an increase in T cell response against cross-reactive sequences between SARS-CoV-2 and other huCoVs after the second dose (**Figure 3D**) with 4/6 participants demonstrated a significant increase in such response (**Supplement 5**). This result suggests that the booster dose activated and enhanced the T cell responses against other huCoVs strains (priming at day 20 did not enhance this response, data not shown). We further evaluated T cell responses against five different human common viruses peptide pools: CMV, EBV, BKV, Adv 3, and 5 on day 0 and day 34 post-vaccination. As expected, all six participants showed T cell responses to all of these viruses at baseline (day 0 before vaccination), ranging between 87 and 236 SFC due to previous exposure to these viruses (**Figure 3E**). Importantly, we have demonstrated that this T cell response was increased in 4/6 participants on day 34 post-vaccination (**Figure 3E**). These results may suggest the presence of cross-reactive epitopes between SARS-CoV-2 and these five viruses.

### Rapid ex-vivo expansion of spike-specific T cells from BNT162b2 vaccinated donors

**Figure 4:**
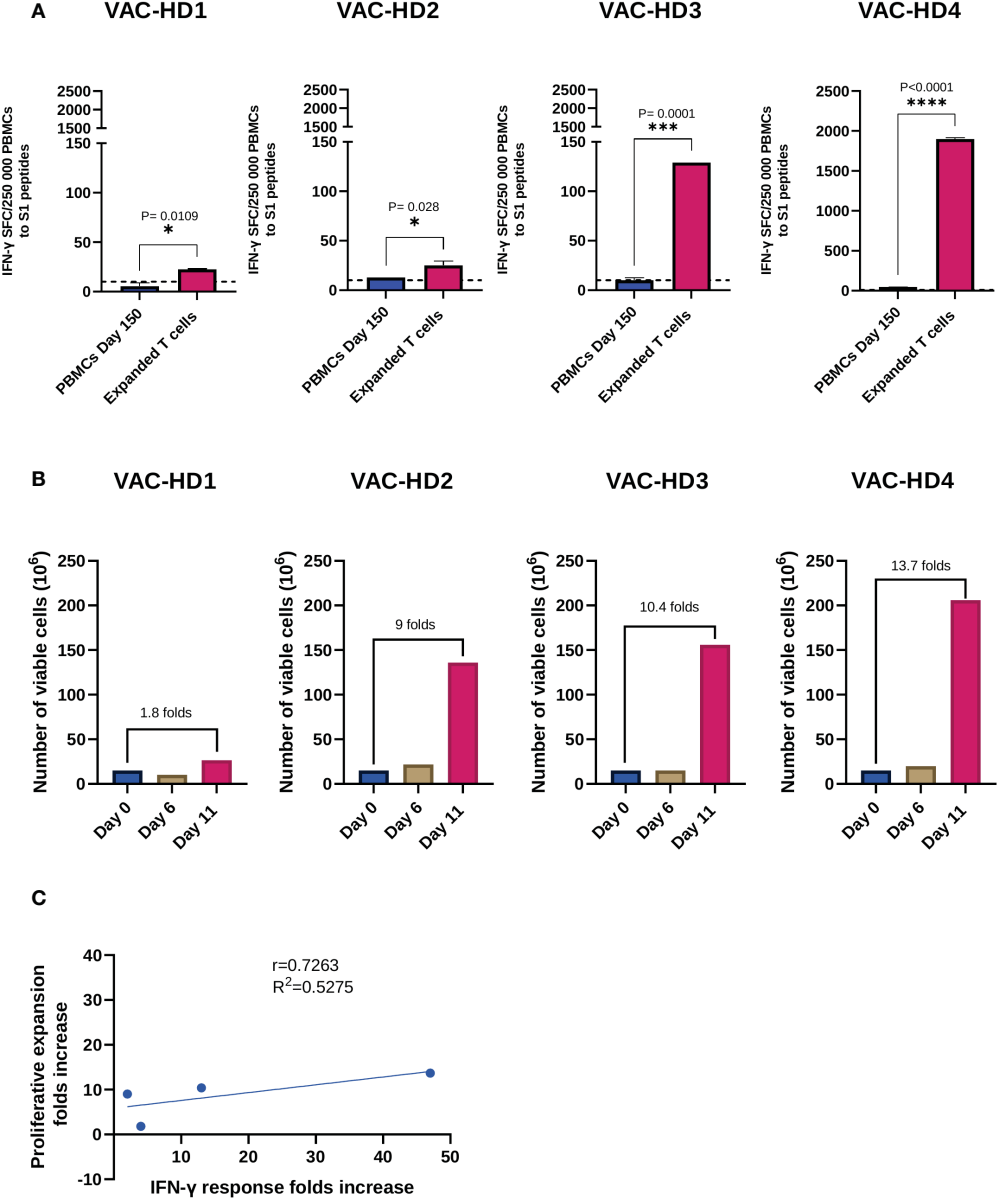
Expansion and functional characterization of expanded spike-specific T cells. **(A)** IFN-γ secretion by pre-expansion PBMCs collected 150 days post-vaccination and spike-specific expanded T cells following S1 peptide pools overnight stimulation. Results are presented as the mean of the number of spots forming cells (SFCs) per 250 000 PBMCs subtracting the background (negative control) count. A positive response was defined as an SFCs of 10 or more. Student t test was used, P value was considered statistically significant when *P ≤ 0.05. All samples were run in duplicates. **(B)** Viable cell counts, and fold expansion were assessed on days 0, 6, and 11 of expansion using trypan blue exclusion dye. **(C)** Correlation between IFN-γ response folds increase and the proliferative expansion folds increase between pre-expansion PBMCs and spike-specific expanded T cells in four BNT162b2 vaccinated participants. The dotted lines represent confidence intervals at 95%. The stars present the level of significance. *P < 0.05; **P < 0.01; ****P < 0.0001.

We next investigated whether we could expand the S-specific T cells from BNT162b2 vaccinated donors, five months post-vaccination, using a rapid *ex-vivo* expansion protocol described in **supplement 6** (see **supplementary material**). Briefly, PBMCs collected from four vaccinated donors were stimulated with the S peptide pools and then cultured in the presence of IL-4 and IL-7 for 11 days in the G-Rex 10 culture device. We first examined the S-specific T cell response in these four participants prior to expansion. T-spot results showed a positive S1-specific T cell response ranging from 10 to 46 SFCs in VAC-HD2, 3, and 4 (**Figure 4A**). However, VAC-HD 1 lacked detectable S1-specific T cells (SFCs=6, below the positive cutoff threshold) (**Figure 4A**). Interestingly, the T cells from VAC-HD2, 3, and 4 were expanded up to 9-fold (136x10^6^ cells), 10.4-fold (156x10^6^ cells), and 13.7-fold (206x10^6^ cells), respectively eleven days post-stimulation (**Figure 4B**). The T cells from VAC-HD1 however failed to adequately expand likely due to the low frequency of the S1-specific T cells before expansion (1.8-fold; 26.5x10^6^ cells) (**Figure 4B**). These results indicate that the frequency of pre-existing S-specific T cells may play a major role in the expansion of such T cells.

We next evaluated the specificity of these expanded T cells by measuring IFN-γ secreting T cells in response to S1 stimulation using the T-spot assay, corresponding PBMCs collected 150 days post-vaccination (Pre-expansion PBMCs) were used as a control. Overall, expanded T cells demonstrated an S1-specific IFN-γ production which was significantly higher than the one detected in pre-expanded T cells for all participants (**Figure 4A**). Our data show that this T cell response varied between the four participants and positively correlated with the proliferative expansion fold (r=0.7263, R^2 =^ 0.5275) (**Figure 4C**). Significant increase in the number of IFN-γ SFC, after T cell expansion, was observed in the four participants (**Figure 4A**): VAC-HD1 (4-fold increase from 6-23 SFCs; *P=0.0109), VAC-HD2 (2-fold increase from 13-25 SFCs; *P=0.028), VAC-HD3 (13-fold increase from 10-129 SFCs; ***P=0.0001) and VAC-HD4 (41-fold increase from 47-1900 SFCs; ****P<0.0001) (**Figure 4A**).

### Phenotypic characterization of S-specific expanded T cells

**Figure 5:**
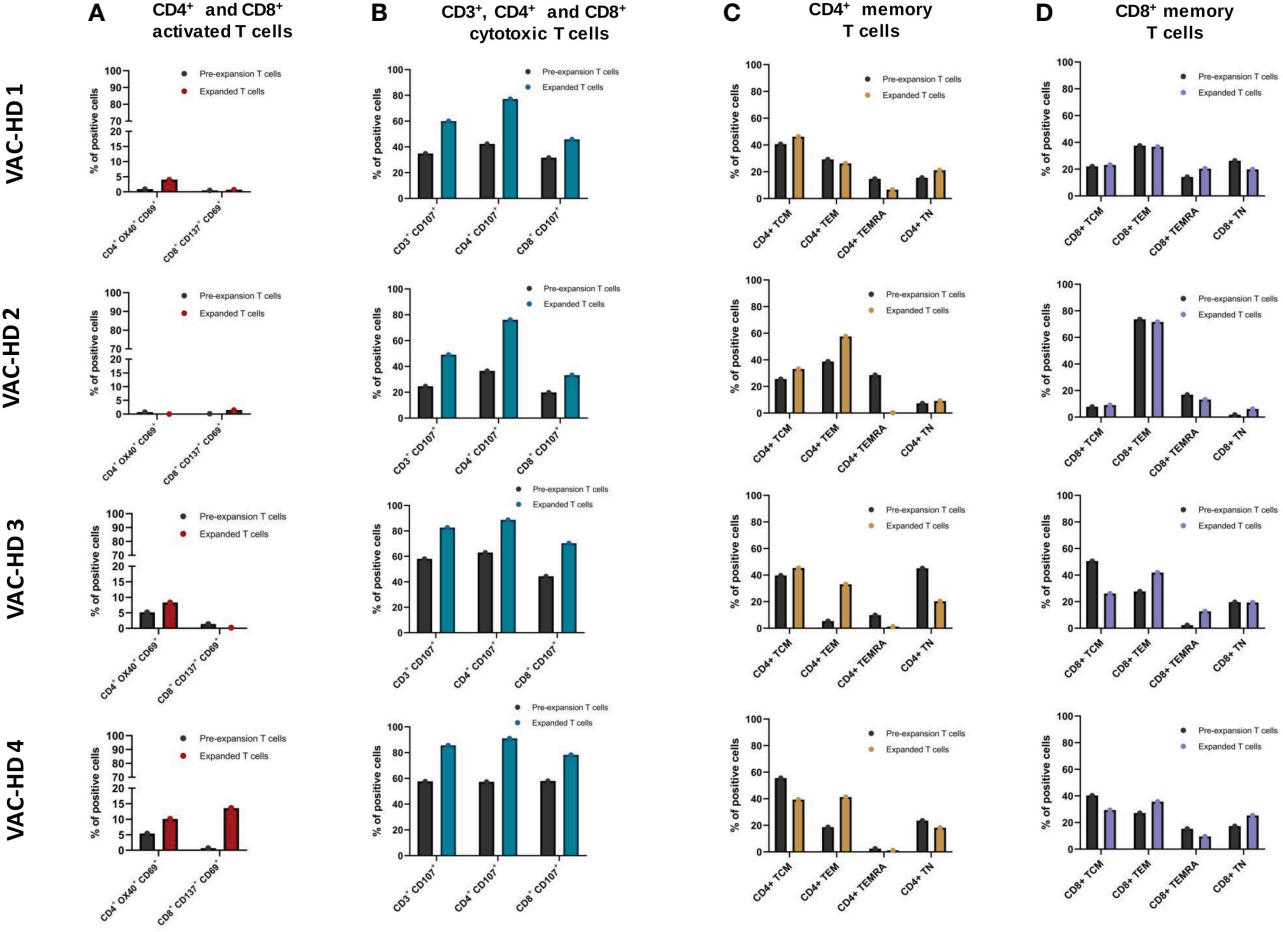
Phenotyping of the spike-specific expanded T cells. **(A)** Frequency of CD4^+^ OX40^+^ CD69^+^ and CD8^+^ CD137^+^ CD69^+^ activated T cells within pre-expansion and expanded T cells following S1 stimulation in four BNT162b2 vaccinated participants. **(B)** Frequency of CD3^+^ CD107^+^, CD4^+^ CD107^+^, and CD8^+^ CD107^+^ cytotoxic T cells within pre-expansion and expanded T cells following S1 stimulation in four BNT162b2 vaccinated participants. **(C)** Frequency of CD4^+^ naïve (CCR7^+^CD45RA^+^), central memory (CCR7^+^CD45RA^−^), effector memory (CCR7^−^CD45RA^−^), and terminally differentiated memory (CCR7^−^CD45RA^+^) within pre-expansion and expanded T cells following S1 stimulation in four BNT162b2 vaccinated participants. **(D)** Frequency of CD8^+^ naïve (CCR7^+^CD45RA^+^), central memory (CCR7^+^CD45RA^−^), effector memory (CCR7^−^CD45RA^−^), and terminally differentiated memory (CCR7^−^CD45RA^+^) within pre-expansion and expanded T cells following S1 stimulation in four BNT162b2 vaccinated participants.

To further analyze the phenotype of the expanded S-specific T cells, the distribution of activated and cytotoxic T cell subsets was assessed using flow cytometry following stimulation with the S1 antigen. We also compared the changes in the distributions of these T cell subsets before and after expansion. The gating strategy and raw data are presented in **supplements 7 and 8** (see **supplementary material**). Overall, the frequencies of both pre-expanded S-specific activated CD4^+^ (CD4^+^OX40^+^CD69^+^) and CD8^+^ (CD8^+^CD137^+^CD69^+^) T cells were higher in VAC-HD3 and 4 compared to VAC-HD1 and 2 (**Figure 5A**). In this, the frequency of S-specific activated CD4^+^ T cells was 0.93% and 0.73% in VAC-HD1 and 2 versus 5.15% and 5.39% in VAC-HD3 and 4 (**Figure 5A**). Whereas the frequency of S-specific activated CD8^+^ T cells was 0.51% and 0.15% for VAC-HD1 and 2 versus 1.39% and 0.7% for VAC-HD3 and 4 (**Figure 5A**). These results indicate that the frequency of pre-existing S-specific T cells prior to expansion was higher in VAC-HD3 and 4 than in VAC-HD1 and 2 which can explain the fact that the expansion was more efficient for these two cases compared to others (**Figure 4B**). Interestingly, an increase in the frequencies of expanded CD4^+^ and/or CD8^+^ S-activated T cells was recorded in all participants when compared to the pre-expanded population (**Figure 5A**). We also showed that induction of the T cell activation markers against the S antigen was higher in the CD4^+^ T cells for VAC-HD1 and VAC-HD3 (4.04% and 8.32% respectively) and in the CD8^+^ T cells for VAC-HD 2 and VAC-HD4 (1.49% and 13.6% respectively) (**Figure 5A**).

We next examined the overall changes in CD3^+^, CD4^+^, and CD8^+^ cytotoxic T cell frequencies within the S-specific expanded T cells compared to the pre-expanded T cells. Expanded T cells presented higher frequencies of CD3^+^CD107^+^, CD4^+^CD107^+^, and CD8^+^CD107^+^ cytotoxic T cells compared to the pre-expanded population in all four participants (**Figure 5B**). In this, the frequencies of CD3^+^CD107^+^ increased from 24.6-58.1% (Pre) to 49.1-85.7% (Post), CD4^+^CD107^+^ from 36.5-63% (Pre) to 76.1-91.1% (Post), and CD8^+^CD107^+^ from 19.9-58% (Pre) to 33.3-78.3% (Post) in all the four participants (**Figure 5B**). Interestingly, the cytotoxic T cell marker CD107^+^ was higher in the CD4^+^ expanded T cell population (76.1% to 91.1%) when compared to the CD8^+^ counterparts (31.6% to 78.3%) in all four participants (**Figure 5B**).

In the next step, we examined the distribution of memory T cell subsets in the expanded T cell population. Based on phenotypic markers, T cell subsets can be classified into four subsets: naïve (CCR7^+^CD45RA^+^), central memory (CCR7^+^CD45RA^−^), effector memory (CCR7^−^CD45RA^−^), and terminally differentiated memory (CCR7^−^CD45RA^+^). The S-specific expanded CD4^+^ and CD8^+^ T cells were found to have different distributions of naïve (TN), effector (TEM), central memory (TCM), and terminally differentiated memory (TEMRA) phenotypes in all four participants (**Figures 5C, D**). For CD4^+^ expanded T cells, the TCM subset was dominant in VAC-HD1 and 3 whereas the TEM subset was aberrant in VAC-HD2 and 4 (**Figure 5C**). However, for CD8^+^ expanded T cells, the TEM subset was dominated in all four participants with a frequency ranging from 35.7 to 71.6% (**Figure 5D**). Interestingly, we demonstrated that VAC-HD1 and 2, who showed the highest frequencies of pre-expanded CD4^+^ TEMRA (**Figure 5C**), had lower S-specific pre-expanded CD4^+^ activated T cells (CD4^+^OX40^+^CD69^+^) frequencies in the compared to VAC-HD3 and VAC-HD4 (**Figure 5A**). Moreover, the frequency of the CD4^+^ TEMRA subset was greatly reduced after T cell expansion in all 4 participants (**Figure 5C**). Importantly, we showed that participants who had a CD4^+^ TCM (CCR7^+^CD45RA^−^) dominance (VAC-HD1, 3 and 4) (**Figure 5C**) expressed high CD69 activation marker in the CD4^+^ activated T cells among the expanded T cells (**Figure 5A**).

## Discussion

Most of the investigations designed to assess the efficacy, immunogenicity, and protective immunity induced by the BNT162b2 vaccine were based on large cohort studies. However, despite their advantages, these studies are providing only a general observation of the vaccine-induced immune response without dissecting such immune responses that is laborious and expansive to investigate in large cohorts. In the present work, we dissected BNT162b2 vaccine-induced humoral and cellular responses in a small cohort study that includes recipients receiving two doses of this vaccine. Our results showed a broad range of variation in both humoral and cellular responses. This interpersonal variation was observed at three stages: the induction was detected on day 20, the peak response reached on day 34, and the response declined 150 days post-vaccination. The factors involved in the interindividual variation in the human immune response to BNT162b2 vaccine are still largely unknown. Previous reports suggest that the interindividual diversity of the human immune responses to common pathogens and vaccines is determined by intrinsic (age and gender), extrinsic (environment), and genetic factors such as genes coding for human leukocyte antigen alleles, major histocompatibility complex molecules, Toll-like receptors and cytokines (36, 37). Recently, Ward et al. identified age and gender as important determinants of humoral response to BNT162b2 vaccine (38). They showed that antibody positivity was higher in females and the youngest age group (38). Although our study was carried out in a small cohort, we have also shown that females had stronger S1-specific T cells and antibody responses compared to male participants.

Consistent with previous reports (39–41), our results also showed that the dynamics of anti-S binding and neutralizing antibodies responses followed the same trend, where they were induced 20 days post-priming, significantly increased after boosting, then declined five months post-vaccination. The peak level of antibody response detected on day 34 after boosting was associated with an increase of 4-fold in total anti-S binding IgG levels but only a 2-fold increase in the neutralizing activity compared to day 20. Our result is in agreement with a recent study where they showed that the immune response induced at the time of peak response following BNT162b2 vaccination was characterized by a high ratio of non-neutralizing antibodies (42) that may confer protection against SARS-CoV-2 infection (43, 44). Interestingly, we were able to show that all six participants maintained the anti-S IgG binding antibody response five months post-vaccination, whereas only four of them maintained the neutralizing activity response.

Recent studies have demonstrated that neutralizing antibody titers are the most critical parameters for vaccine efficacy evaluation and prediction of SARS-CoV-2 protective immunity (5–7). Many efforts have been made to develop high throughput assays for neutralizing antibody detection that can surrogate the classical neutralizing cell-based assays that require specific laboratory facilities, skilled personnel, and a long (2-4 days) execution time (8, 9). We investigated in this study whether a novel simple automated assay (Jess) could surrogate the neutralization assay for estimation of neutralizing activity. Jess is a robust anti-SARS-CoV-2 binding antibodies surveillance test, which is simple, fully automated, rapid, and can be easily used in laboratories without the need for BSL3 facilities. Using this assay, we have shown that only anti-S IgG (antibodies against the whole spike antigen) had a strong positive significant correlation (r=0.863, R^2 =^ 0.7448, P <0.0001) with the anti-S neutralizing activity compared to other SARS-CoV-2 spike domain-specific IgG (S1-RBD, S1, and S2). Our results are in agreement with two very recent studies that used a chemiluminescent immunoassay for the quantitative determination of SARS-CoV-2 IgG binding antibodies (45, 46). The first study reported a linear correlation between anti-S IgG and surrogate neutralizing antibody levels for wild-type SARS-CoV-2 and variants of concern (VOCs) in BNT162b2 vaccinated and recovered health care workers (45). the second study also showed a strong correlation (R^2 =^ 0.72) between the anti-S antibody IgG titers detected by a chemiluminescent immunoassay and surrogate neutralizing activity (46). Taken together, our results indicate that Jess provides a robust anti-S neutralizing activity surveillance/prediction test.

The characterization of the T cell response in BNT162b2 vaccinated individuals indicated that a single dose of vaccine was not able to induce spike-specific T cell response in 30% of the participants indicating the necessity of a booster dose for efficient and durable protection (47). Similar to this, our results showed that 33% of vaccinated participants lacked a detectable spike-specific T cell response after one dose of this vaccine. However, the second dose was able to induce the spike-specific T cell response in 100% of the participants with a median increase of 3-fold in the T cell response frequencies compared to that observed after priming. Importantly, we demonstrated that the booster dose was able to also activate and enhance T cell responses against other huCoVs suggesting the presence of cross-reactive epitopes between SARS-CoV-2 and other huCoVs. Our findings are supported by a recent study showing the presence of common epitopes between SARS-CoV-2 and huCoVs (48). We have also demonstrated that pre-existing memory T cells, naturally induced during past infections of our participants with the five human common viruses (CMV, EBV, Adv 3 and 5, BKV), were *in vivo* expanded following the BNT162b2 vaccination. This suggests the presence of cross-reactive epitopes between SARS-CoV-2 and these five viruses derived-antigens. Indeed, the sequence similarity between EBV and SARS-CoV-2 has been well established (49) however, no study related to sequence or epitope similarity for the other viruses have been investigated. Furthermore, our data showed the persistence of spike-specific T cell response five months post-vaccination in 67% of the participants.

BNT162b2 mRNA vaccine clinical trial showed 95% effectiveness in preventing SARS-CoV-2 infection (3). This trial predominantly excluded patients with immunocompromising conditions (50), which present 2% of the global population (51). Indeed, recent studies have demonstrated that a two doses vaccine regimen does not produce sufficient strong immune responses and protection in immunocompromised patients and elderly people (31–33). Considering the ineffectiveness of current SARS-CoV-2 antibody-based immunotherapy due to the development of novel mutations and the immune escape of the VOCs (52), other therapeutic options are warranted. Adoptive SARS-CoV-2 specific T cell therapy represents an attractive therapeutic option in which viral immune escape is likely to be avoided as the recognized T cell epitopes are well conserved among the emerging SARS-CoV-2 variants (25). Moreover, adoptive cellular therapy with *ex-vivo* expanded specific T cells against other viruses (CMV, EBV, Adv, HHV6, and BKV) has been demonstrated to have efficacy in combating severe viral diseases in patients with immunodeficiency (53). In addition, studies in animal models have also shown that adoptive therapy with CD4^+^ and/or CD8^+^ T cells can efficiently control respiratory infections including SARS-CoV-1, MERS, and influenza viruses (54–56). Here, we adapted a rapid *ex-vivo* T cell expansion protocol for spike-specific T cell expansion to be potentially used for adoptive-cell therapy in severe COVID-19, immunocompromised individuals, and other high-risk groups. In this protocol, we expanded spike-specific T cells from vaccinated donors isolated five months post-vaccination to avoid spike-specific T cells exhaustion during the e*x-vivo* expansion and to mimic the *in vivo* stimulation and boosting effect of the booster dose which is usually given between five to six months post-priming. Moreover, the long-term persistence of memory T cells following vaccination or viral infection has been well reported (57, 58). The response mediated by such memory T cells, upon re-exposure to the antigen, is more rapid and effective than the primary response (59). In this rapid protocol (11 days) we were able to expand T cells up to 11-folds in 3/4 participants. A minimum S-specific T cell number ≥10 SFCs was required to support T cell expansion. Moreover, participants who had a higher frequency of pre-expanded S-specific activated CD4^+^ (CD4^+^OX40^+^CD69^+^) and CD8^+^ (CD8^+^CD137^+^CD69^+^) T cells tended to have a higher expansion rate of S-specific T cells. The specificity of expanded T cells was measured with a newly modified T-spot assay in which the high-homology peptide sequences cross-reactive with other coronaviruses were removed. Expanded T cells demonstrated a significant increase of S1 spike-specific IFN-γ producing cells compared to the pre-expanded T cells for all participants. Interestingly, these S-specific expanded T cells had higher frequencies of both activation and cytotoxic markers important for viral clearance after re-exposure (60). Adoptively transferring such expanded T cells may be used as an attractive approach to restore and/or boost the cytotoxic T cell response in severe COVID-19, immunocompromised patients and elderly people with impaired cytotoxic T cell response to SARS-CoV-2 (28, 31–33, 61). Interestingly, the cytotoxic T cell marker CD107^+^ was higher in the CD4^+^ expanded T cell population when compared to the CD8^+^ counterparts in all four participants. This result indicates that CD4^+^ cytotoxic T cells play a major role in the S-specific cell-mediated cytotoxic response following BNT162b2 immunization. In line with this, it has been reported that S-specific T cell response, elicited by SARS-CoV-2 infection, was dominated by the CD4^+^ subset in COVID-19 patients (62, 63).

It has been well established that central memory (TCM) and effector memory (TEM) T cell subsets have distinct functions and migratory properties (64, 65). Therefore, we examined the distribution of these memory T cell subsets in our spike-specific expanded T cells. There was a variation in the distribution of the CD4^+^ and CD8^+^ memory T cell subsets among all four participants. For CD4^+^ memory T cells, the TCM subset was dominant in 2/4 participants whereas the TEM subset was aberrant in the 2 remaining participants. CD4^+^ TCM resides within the lymphoid organs and are known for their rapid proliferation and production of IL-2 and IL-10 upon restimulation (66). However, CD4^+^ TEM reside in peripheral tissues and exhibit immediate cytokine secretion of IFN-γ and IL-4 upon restimulation (66). For CD8^+^ T cells, the TEM subset was dominated in all four participants. CD8^+^ TEM also resides in peripheral tissue and provides immediate protection following antigenic stimulation by the secretion of perforin (66). Another subset of memory T cells known as terminally differentiated memory (TEMRA) has been demonstrated to exhibit numerous characteristics of immuno-senescence such as defects in proliferation and effector functions (67). Both CD4^+^ and CD8^+^ TEMRA are known to accumulate in the aging process and pathological conditions such as arthritis rheumatoid and persistent viral infection (68–75). Therefore, we investigated this phenomenon in our expanded spike-specific T cells. Interestingly, we found that participants who showed the highest frequencies of CD4^+^ TEMRA cells prior to expansion tended to have lower S-specific activated CD4^+^ T cells and a lower fold increase of the S1-specific IFN-γ response of expanded spike-specific T cells compared to the pre-expanded population. These results suggest that this subset of CD4^+^ TEMRA cells may contribute to the impairment in the S-specific CD4^+^ T cells development and T cell expansion inefficacy. We have also demonstrated that the frequencies of CD4^+^ TEMRA were greatly reduced after S-specific T cell expansion. On the other hand, immuno-senescence associated with defects in immune proliferation and effector functions has been shown to correlate with an increased susceptibility to viral infection and a decreased vaccine immunogenicity (76). Indeed, several studies suggested that influenza vaccine inefficacy in aged individuals can be mainly related the immune system immunosenescence (77, 78). Another recent study has also demonstrated that mRNA COVID-19 vaccine immunogenicity was negatively correlated with the accumulation of T cell expressing signs of immunosenescence (79).

CD69 is widely used as an activation marker for T cells and natural killer cells, however, the precise role of this marker in these immune cells is not yet well elucidated (80). Recent evidence suggests that the expression level of CD69 controls the migration and retention of CD4^+^ memory T cells in their specific niches (81). Similarly, another study suggests that upregulation of CD69, after yellow fever vaccination, can promote T cell migration and retention in the lymph nodes, the home for TCM (80). Therefore, we hypothesized that an increase of the CD69 marker after BNT162b2 vaccination may control the homing and migration of S-specific CD4^+^ TCM. Interestingly, we showed that participants who had a CD4^+^ TCM dominance also expressed a high CD69 activation marker in the CD4^+^ activated T cells among the expanded T cells. Further studies are required to clarify the role of CD69 in vaccine-induced CD4^+^ memory T cell migration and homing.

In conclusion, this is the first pilot study that highlights the interpersonal heterogeneity of the humoral and cellular responses to the BNT162b2 vaccine. We have demonstrated for the first time a strong correlation between Jess and neutralization cell-based assay. we also validated the feasibility and efficacy of a rapid *ex-vivo* spike-specific T cell expansion protocol from BNT162b2 vaccinated individuals that can be used in the future to establish a biobank for adoptive transfer of allogeneic HLA-matched spike-specific T cells as therapeutic and/or prevention options in severe COVID-19, immunocompromised patients, and elderly people. The limitation of our study is the use of a small number of participants and further studies with larger sample sizes are needed to confirm our results.

## Data availability statement

The original contributions presented in the study are included in the article/**Supplementary Material**. Further inquiries can be directed to the corresponding authors.

## Ethics statement

The studies involving human participants were reviewed and approved by Institutional Review Board (IRB) committee of HMC (Project number MRC-01-21-113). The patients/participants provided their written informed consent to participate in this study.

## Author contributions

SD, SM, MM, and VPI: Conceptualized the study, planned, and supervised the experiments. SM: performed experimental work, analyzed data, and generated figures. SM and SD: designed and wrote the manuscript. NT, MM, VPI, MKS, FHA, QF, SH and FS helped in the experimental work. SD, MM, VPI, AR, HMY, FBA, ASO, AAH, MAM, MUA, AWA, SU, LJA, LA, MJ, MG, NA, HMHS, AMJ, MN, AZ, FA and SRA: Critical revision and editing of the scientific contents of the manuscript. All authors read and approved the final manuscript.
