# Supplementary material for: Persistence of spike-specific immune responses in BNT162b2-vaccinated donors and generation of rapid ex-vivo T cells expansion protocol for adoptive immunotherapy: A pilot study
Source: Front Immunol. 2023 Feb 2;14:1061255. doi: 10.3389/fimmu.2023.1061255 (PMC9933868; doi:10.3389/fimmu.2023.1061255)
Supplement: Supplementary file 1 [file DataSheet_1.pdf]

Supplement 1

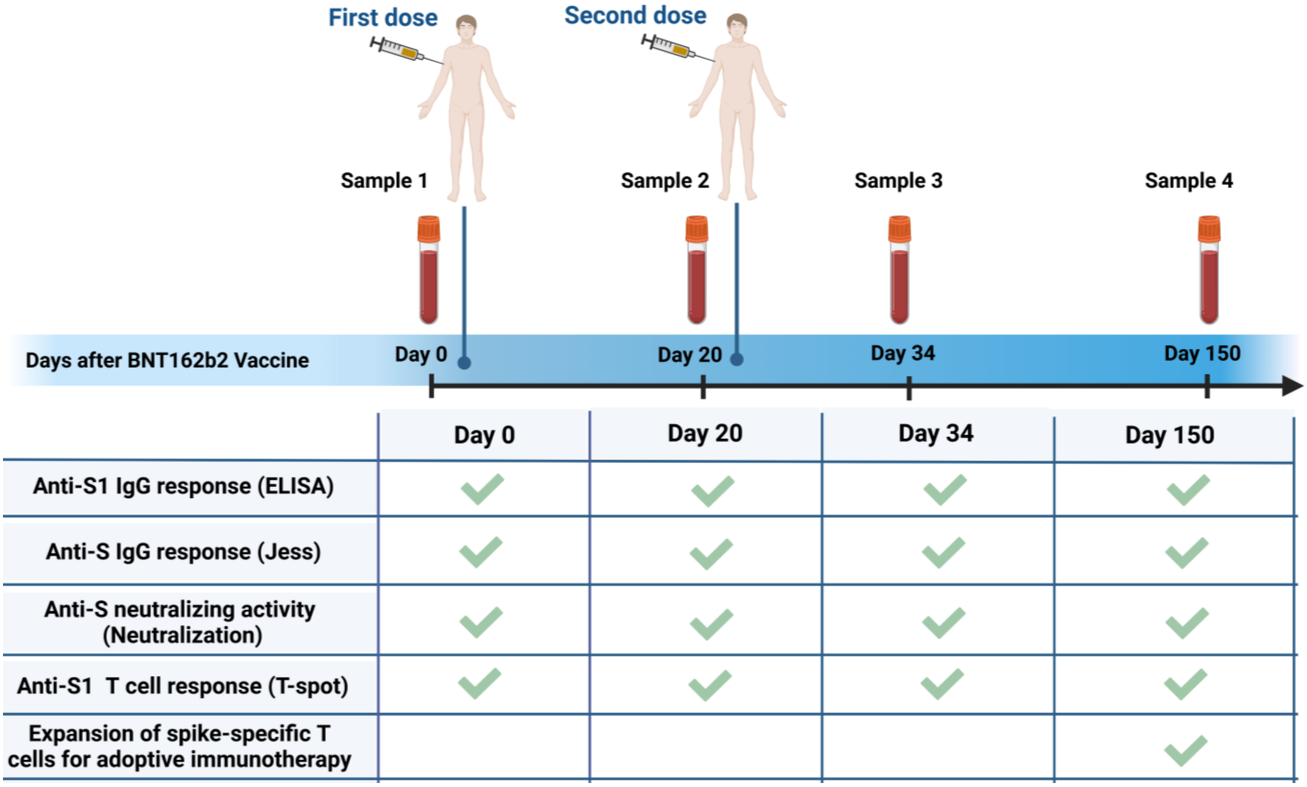

Supplement 1: Vaccination schedule and sample collection. Study participants (n=6) received two doses of the BNT162b2 vaccine. Peripheral blood samples were obtained on day 0 (prior vaccination), day 20 (pre-boost), day 34 (14 days post-boost), and day 150 post-vaccination (5 months after the first vaccination dose). The antibody response elicited by the BNT162b2 vaccine was analyzed using 3 different immunoassays (Jess, ELISA, and Neutralization). The T cell response against S1 peptides and other antigens was assessed using the T-spot assay. 150 days (5 months) post-vaccination, the spike-specific T cells were expanded to be potentially used for adoptive-cell therapy in severe COVID-19, immunocompromised patients, and elderly persons

Supplement 2

Participant code	Gender	Age	Vaccine
**VAC-HD1**	Male	57	BNT162b2 (Pfizer)
**VAC-HD2**	Female	37	BNT162b2 (Pfizer)
**VAC-HD3**	Male	41	BNT162b2 (Pfizer)
**VAC-HD4**	Female	39	BNT162b2 (Pfizer)
**VAC-HD5**	Female	28	BNT162b2 (Pfizer)
**VAC-HD6**	Female	29	BNT162b2 (Pfizer)

Supplement 2: Participant's demographic characteristics

Supplement 3

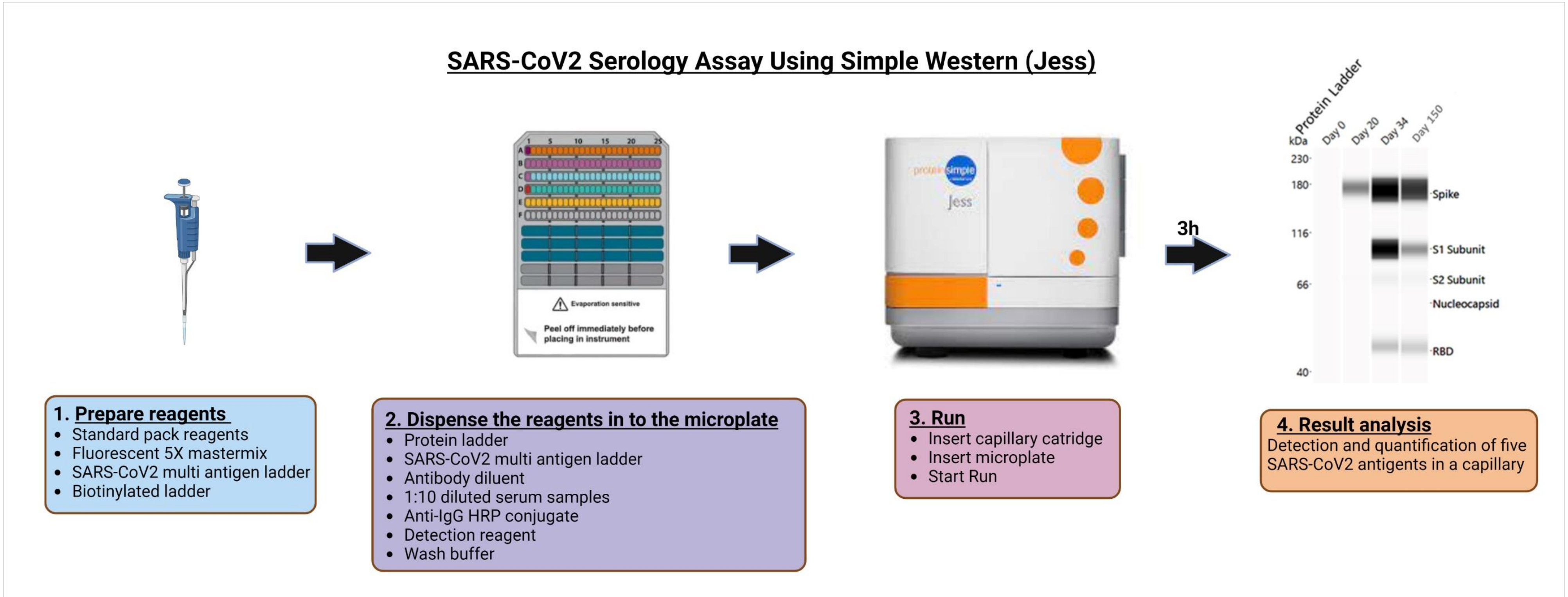

Supplement 3: The working principle of the SARS-CoV2 Jess Simple Western serology assay. SARS-CoV-2 proteins were run in capillaries. The SARS-COV-2 specific human antibodies (reactive to S1-RBD, S1, S2, S, and N) present in the sera samples serve as primary antibodies that were then detected with anti-goat HRP-conjugated anti-human IgG antibodies The chemiluminescent revelation was established with peroxide/luminol-S. The digital image of the capillary chemiluminescence was captured with Compass Simple Western software that automatically calculated the area of the signal (chemiluminescence intensity).

Supplement 4

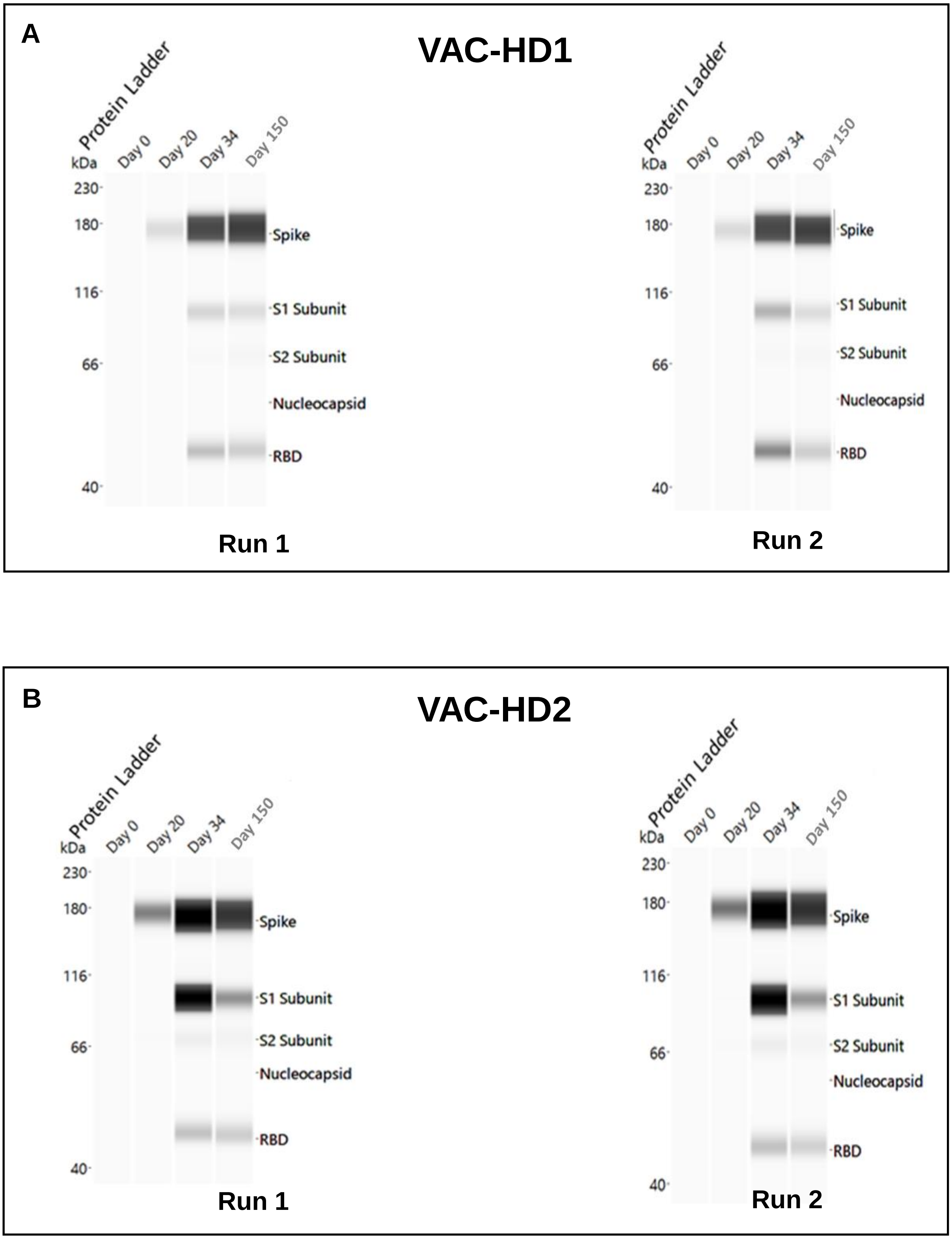

Supplement 4

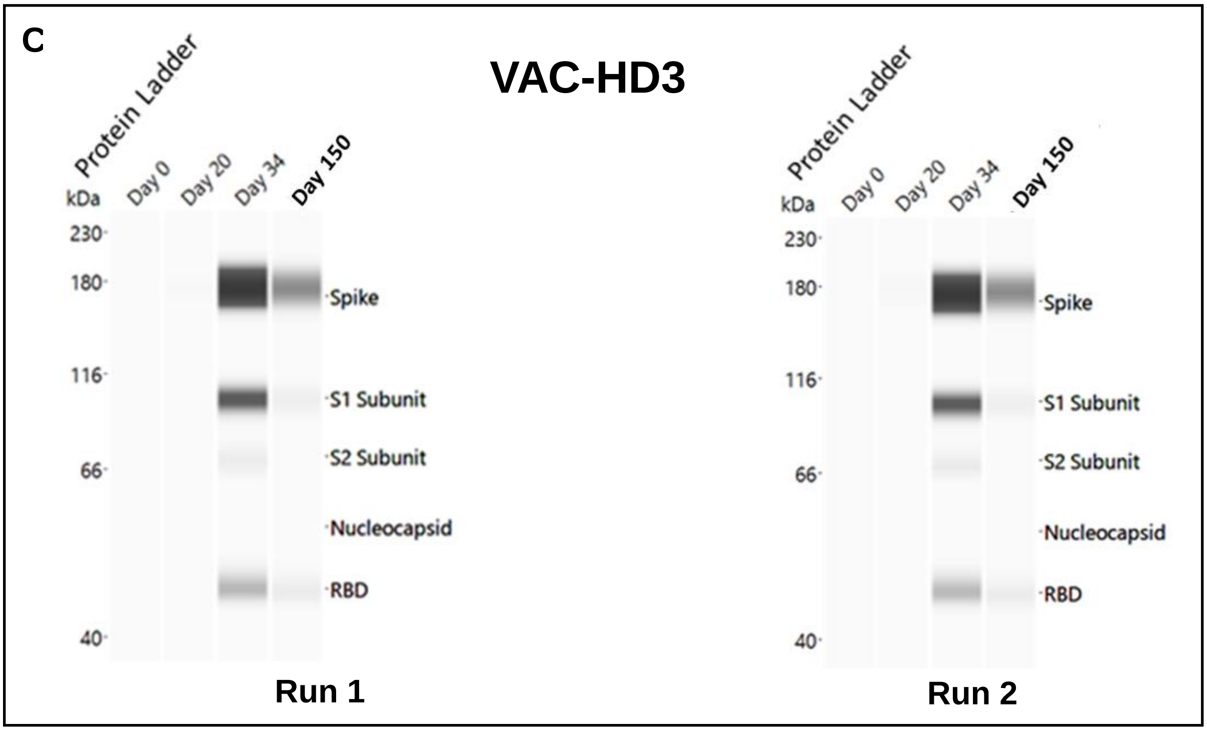

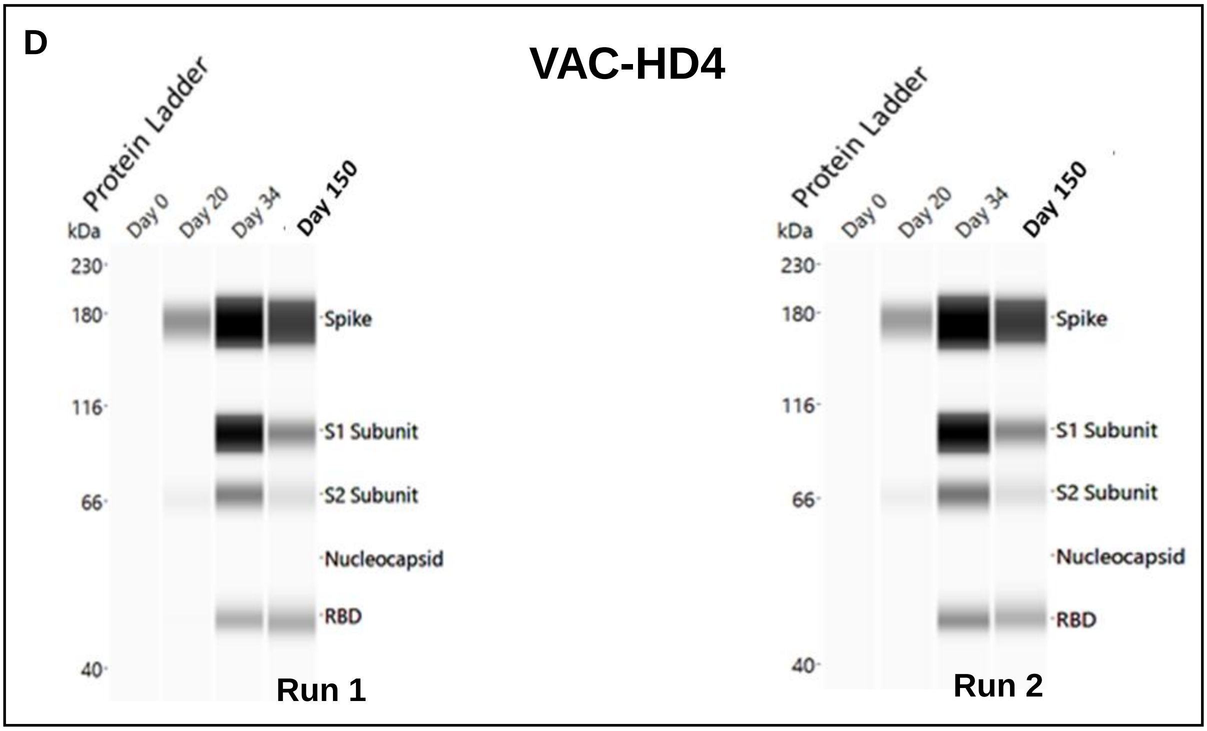

Supplement 4

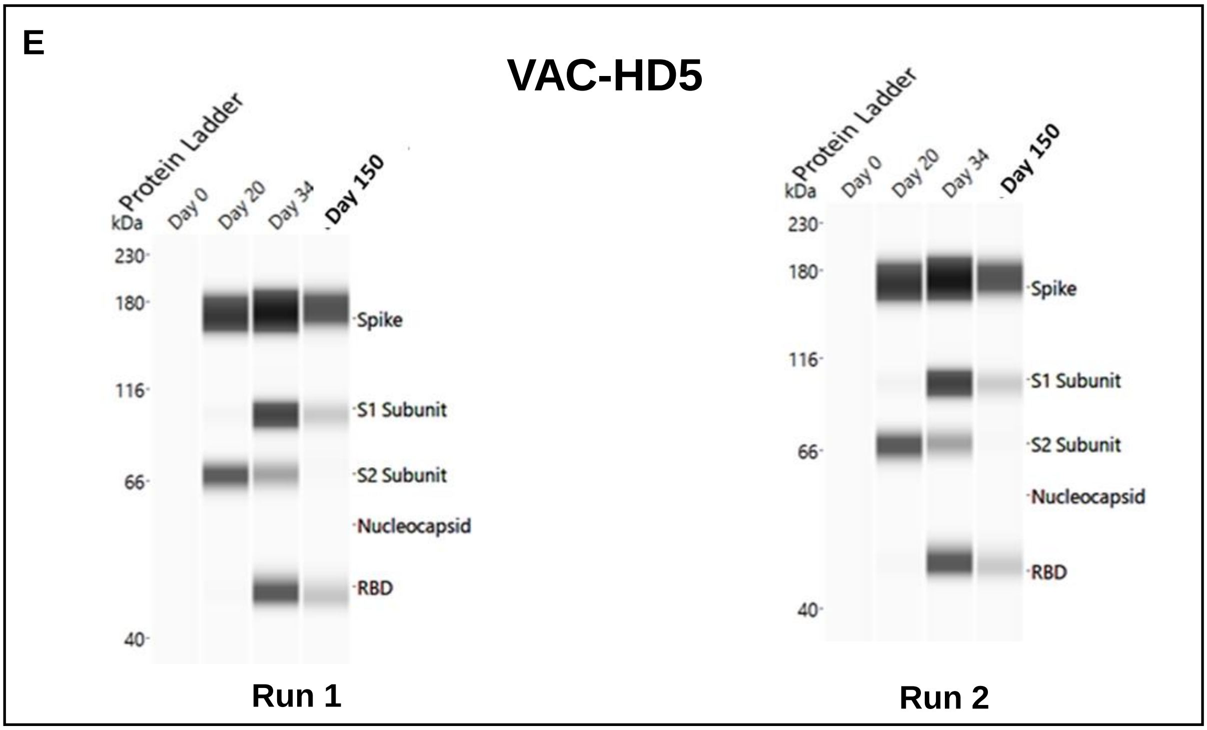

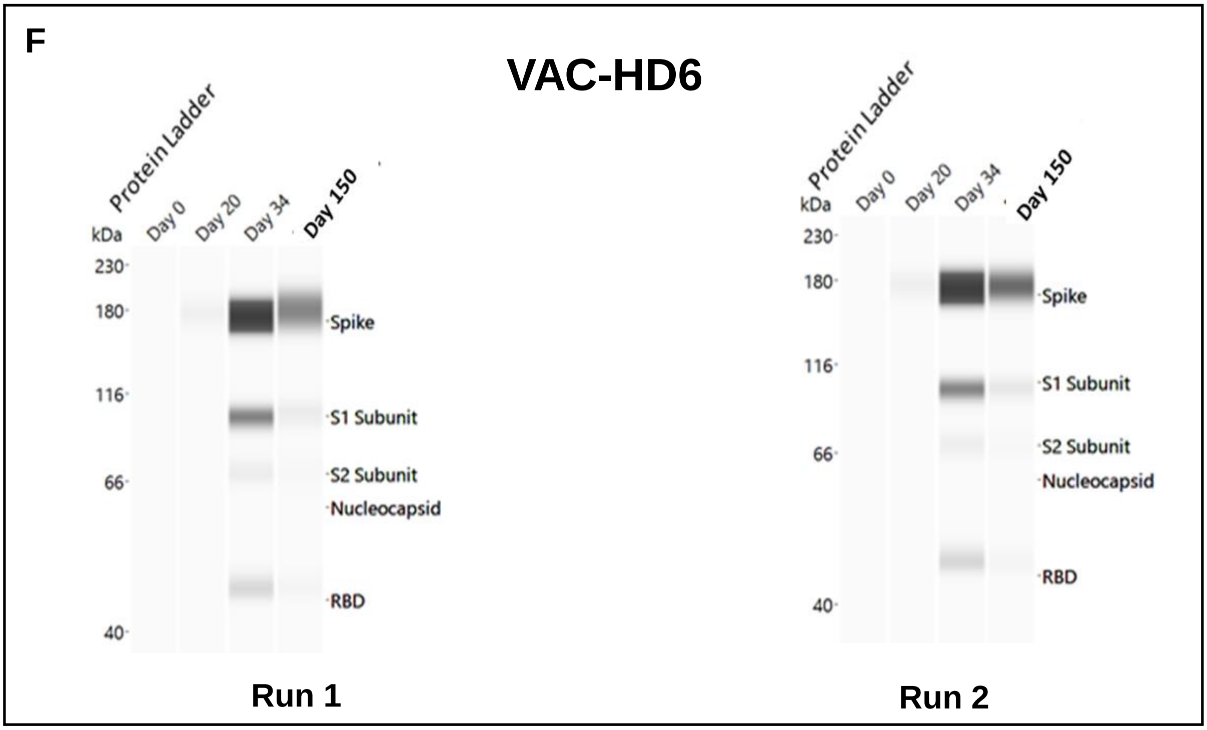

Supplement 4: Detection of SARS-COV-2 specific antibodies system in BNT162b2 vaccinated participants using Jess Simple Western. Sera samples were collected from six BNT162b2 vaccinated participants (VAC-HD1 to VAC-HD6) at the baseline, 20-, 34-, and 150-days post-vaccination. Human IgG antibodies reactive to 5 viral antigens: S1-RBD, S1, S2, S, and N were detected in sera samples using Jess. The digital image of the capillary chemiluminescence of each sample is presented in these graphs.

Supplement 5

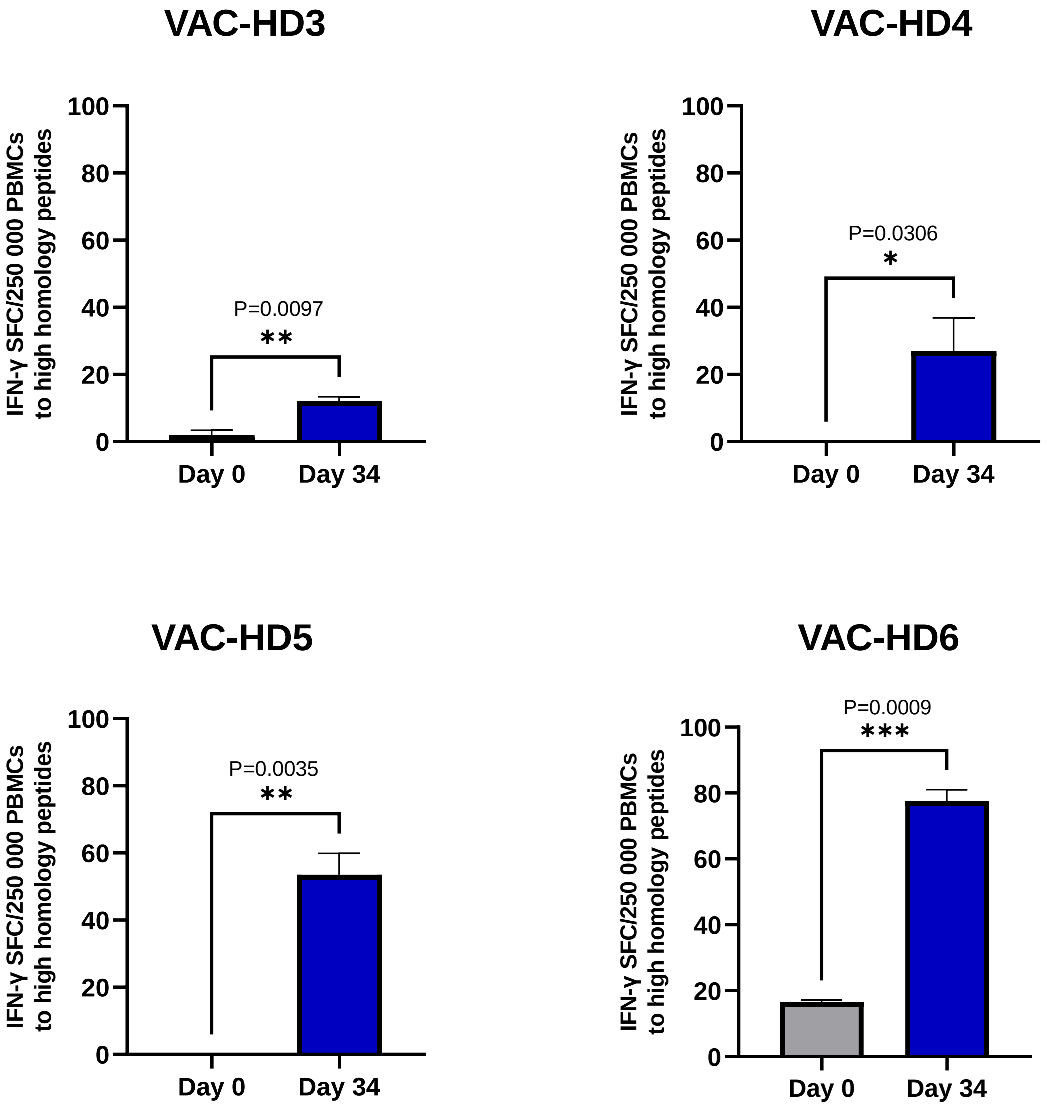

Supplement 5: T cell response to high homology peptide pools in four BNT162b2 vaccinated participants. The T cell response to high homology peptide pools was evaluated on day 0 and day 34 post-vaccination. 4/6 participants (VAC-HD 3, 4, 5, and 6) demonstrated a significant increase in this response.

Supplement 6

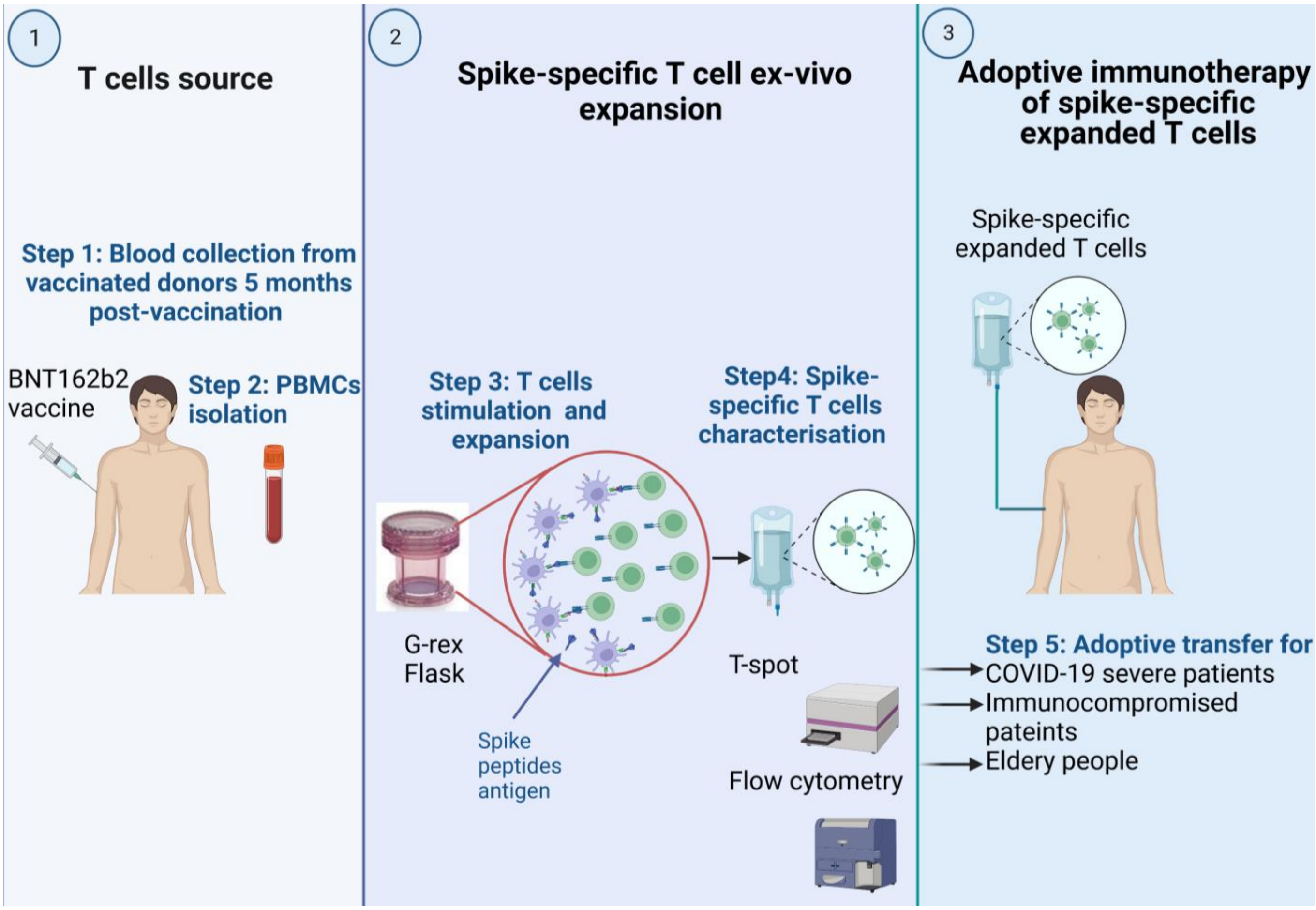

Supplement 6: Rapid *ex-vivo* T cells expansion protocol for adoptive immunotherapy. PBMCs collected from four vaccinated donors five months post-vaccination were stimulated with the S peptide pools and then cultured in the presence of IL-4 and IL-7 for 11 days in the G-Rex 10 culture device. Cells were counted on day 6 and fresh culture media with cytokines was added. Cells were harvested and evaluated for antigen specificity (using T-spot assay) and functionality (using flow cytometry) on day 11 of expansion. The expanded spike-specific response showed higher frequencies of both activation and cytotoxic markers. Adoptively transferring such expanded T cells may be used as an attractive approach to restore and/or boost the cytotoxic T cell response in severe COVID-19 patients and vaccinated elderly/immunocompromised patients with impaired cytotoxic T cell response to SARS-COV-2.

Supplement 7

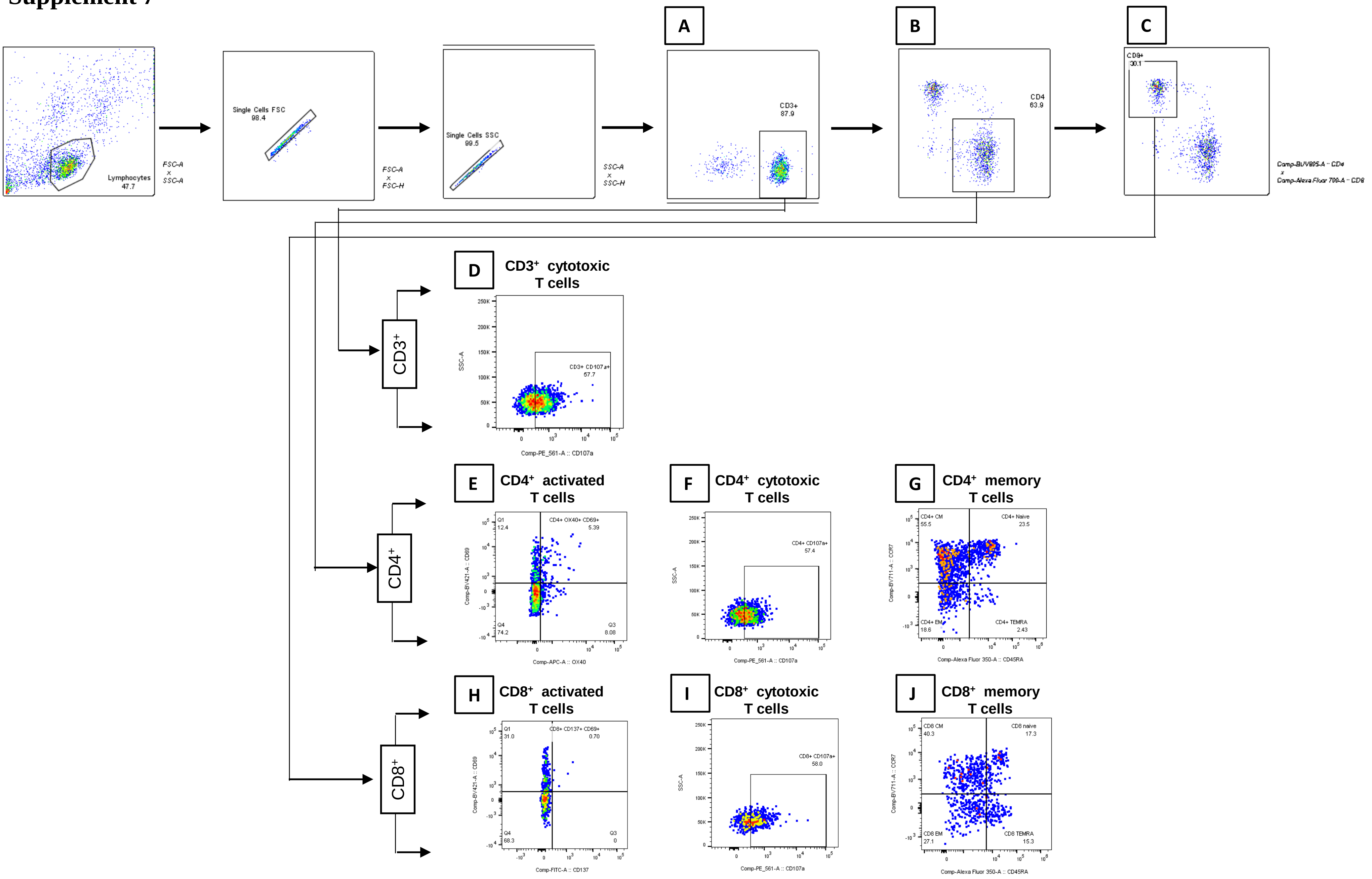

Supplement 7: Gating strategy to identify the principal $CD4^+$ and $CD8^+$ T cell subsets, and other T cell subpopulations. Color dot plot of a representative subject. **(A)** T cells were identified and electronically gated on orthogonal light scatter signals and CD3 immunopositivity. **(B and C)** Then $CD3^+$ $CD4^+$ and $CD3^+$ $CD8^+$ T cells were identified. **(D)** Gating on $CD3^+$ T cells, cytotoxic $CD3^+$ T cells were identified as positive $CD3^+$ $CD107^+$ cells. **(E)** Gating on $CD3^+$ $CD4^+$ T cells, activated $CD4^+$ T cells were identified as positive $CD4^+$ $OX40^+$ $CD69^+$ cells. **(F)** Gating on $CD3^+$ $CD4^+$ T cells, cytotoxic $CD4^+$ T cells were identified as positive $CD4^+$ $CD107^+$ cells. **(G)** Gating on $CD3^+$ $CD4^+$ T cells, the memory $CD4^+$ T cell subsets were identified based on CCR7 and CD45RA expression: naïve (TN, $CD4^+$ $CCR7^+$ $CD45RA^+$), central memory (TCM, $CD4^+$ $CCR7^+$ $CD45RA^–$), effector memory (TEM, $CD4^+$ $CCR7^–$ $CD45RA^–$), and terminally differentiated memory (TEMRA , $CD4^+$ CCR7 $^–CD45RA^+$). **(H)** Gating on $CD3^+$ $CD8^+$ T cells, activated $CD8^+$ T cells were identified as positive $CD8^+$ $CD137^+$ $CD69^+$ cells. **(I)** Gating on $CD3^+$ $CD8^+$ T cells, cytotoxic $CD8^+$ T cells were identified as positive $CD8^+$ $CD107^+$ cells. **(J)** Gating on $CD3^+$ $CD8^+$ T cells, the memory $CD8^+$ T cell subsets were identified based on CCR7 and CD45RA expression: naïve (TN, $CD8^+$ $CCR7^+$ $CD45RA^+$), central memory (TCM, $CD8^+$ $CCR7^+$ $CD45RA^–$), effector memory (TEM, $CD8^+$ $CCR7^–$ $CD45RA^–$), and terminally differentiated memory (TEMRA, $CD8^+$ CCR7 $^–CD45RA^+$)

Supplement 8

VAC-HD1

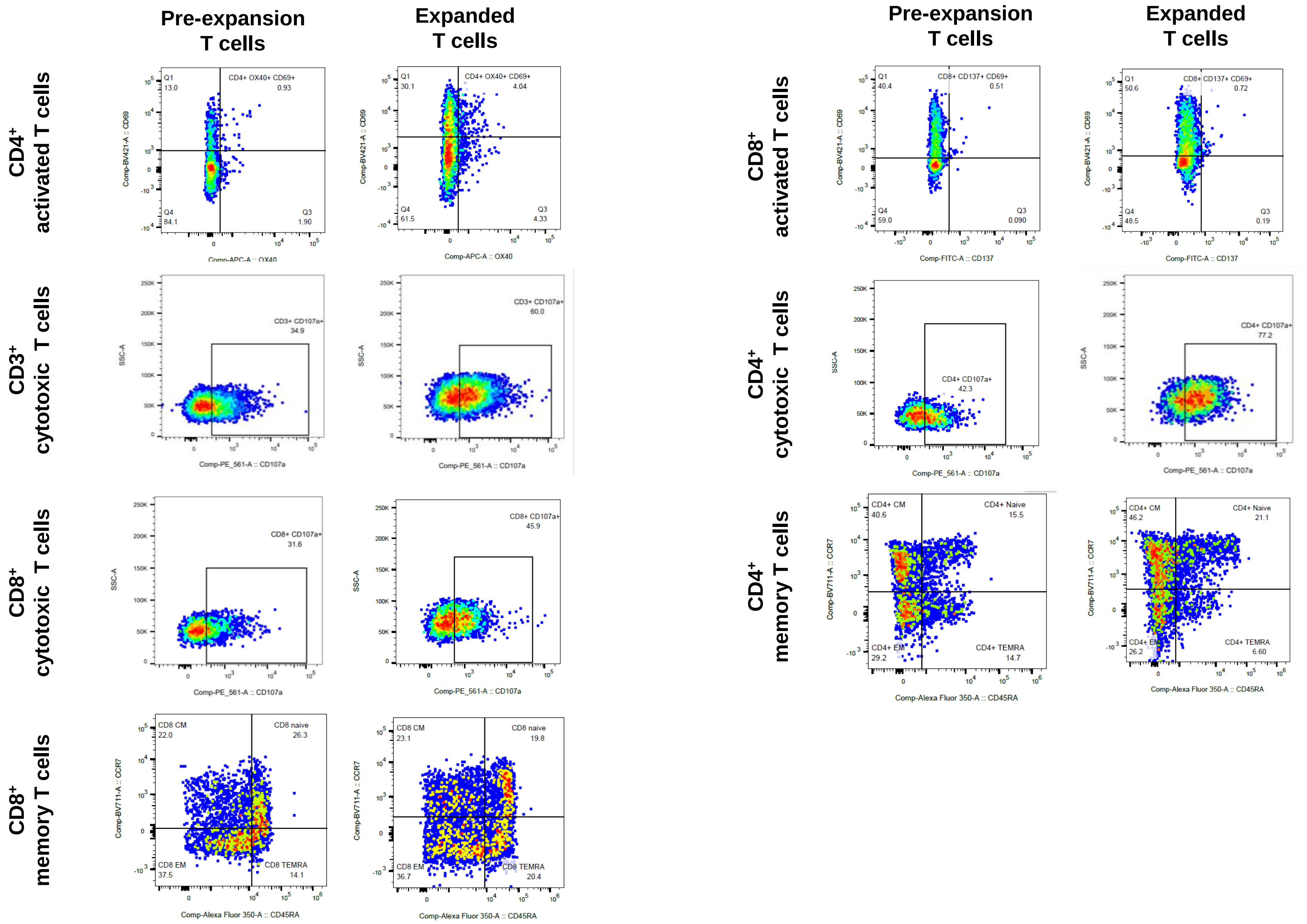

Supplement 8

VAC-HD2

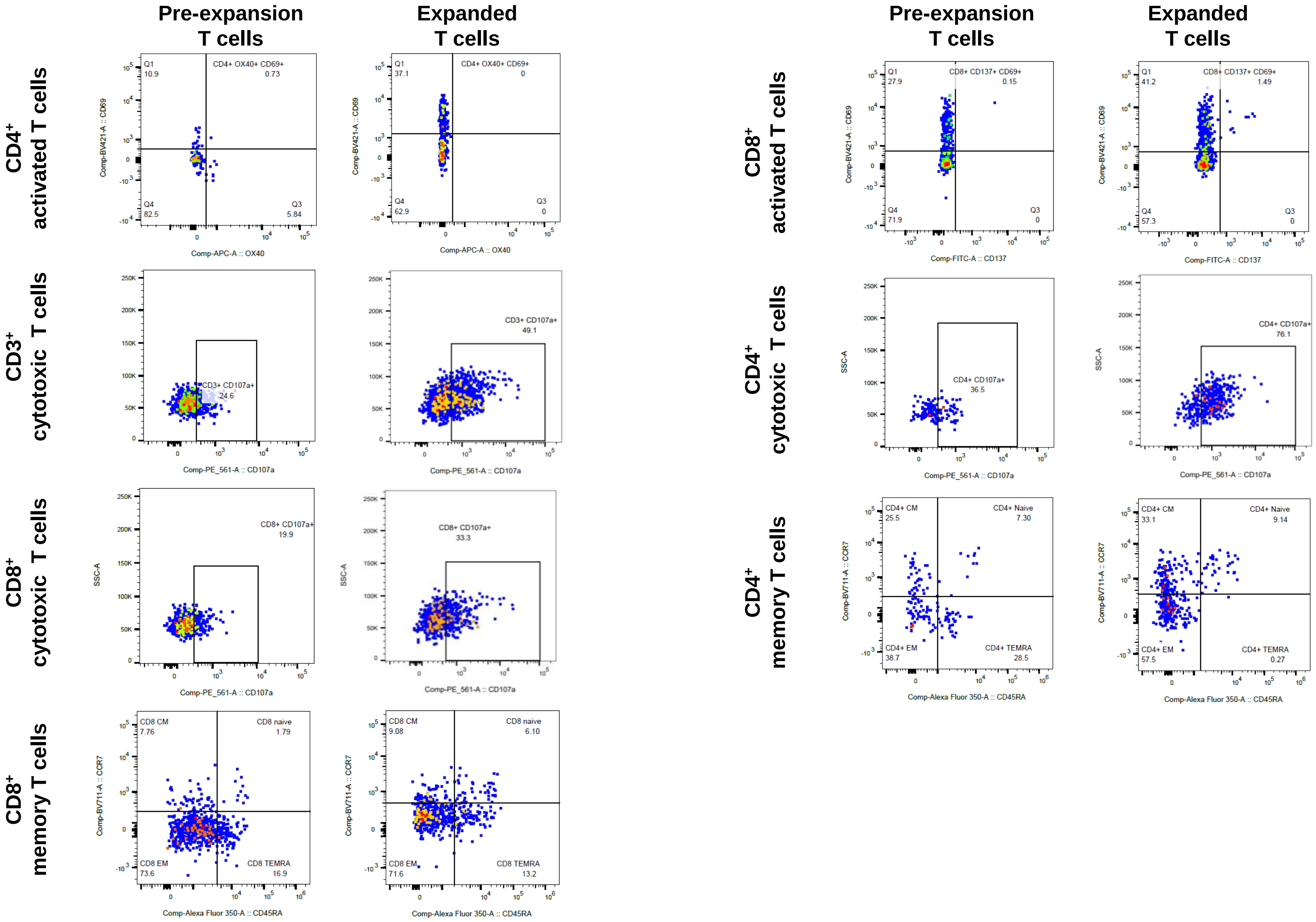

Supplement 8

VAC-HD3

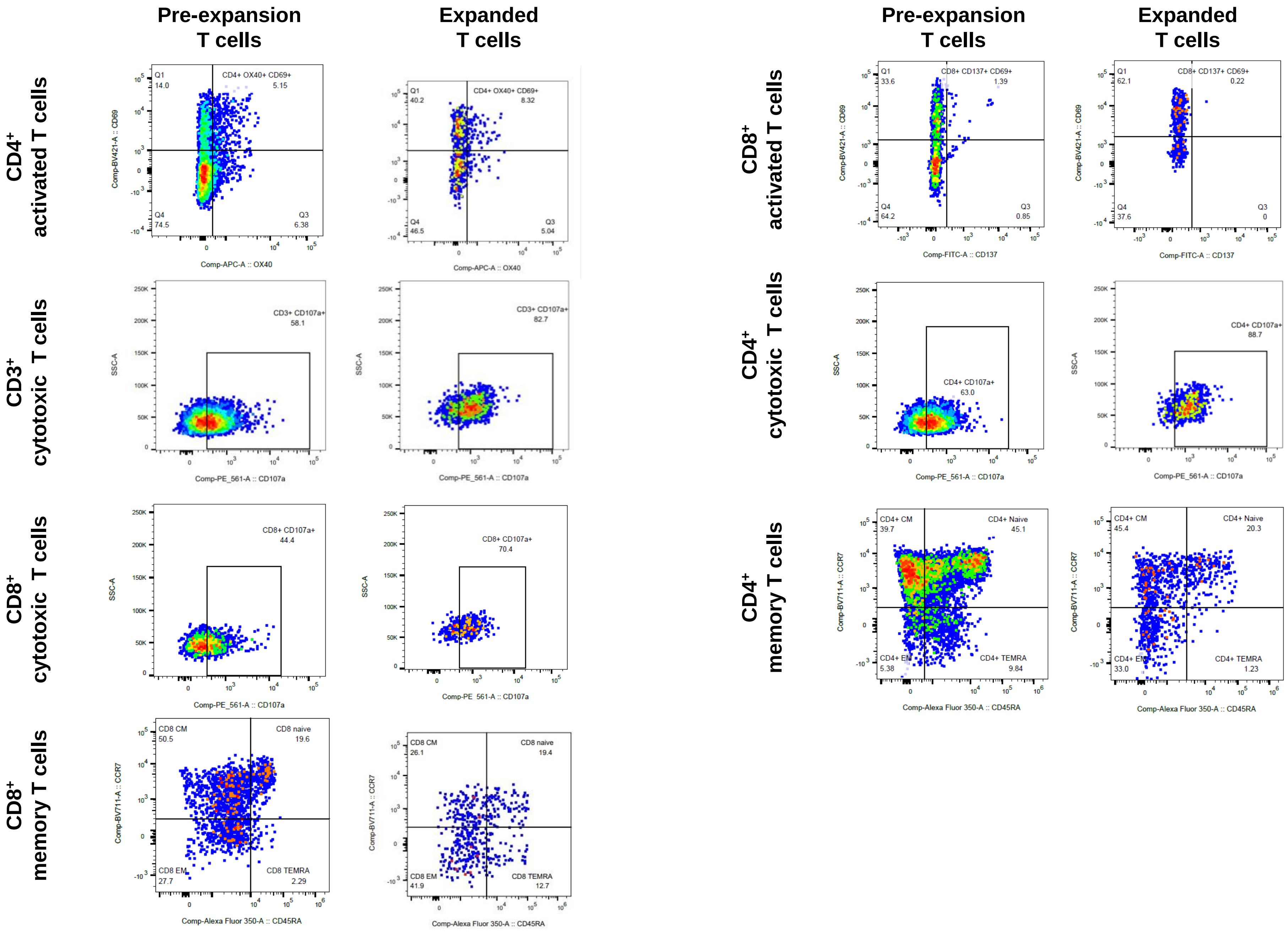

Supplement 8

VAC-HD4

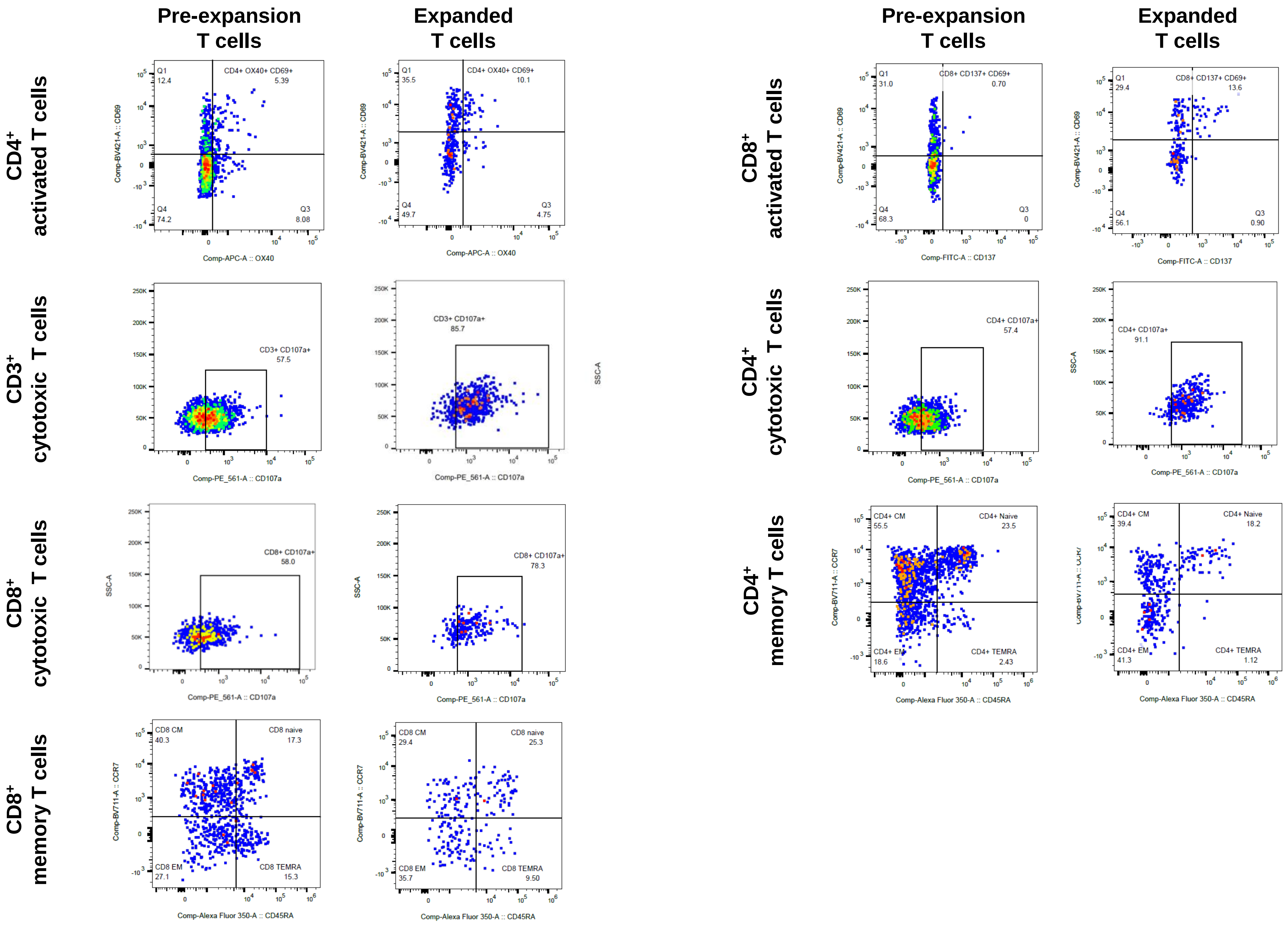

Supplement 8: Phenotypical characterization of the spike-specific expanded T cells in four BNT162b2 vaccinated participants (VAC-HD1, 2, 3, and 4). The histograms presented in figure 5 summarize the results presented in supplement 8. First, the Frequencies of $CD4^+$ $OX40^+$ $CD69^+$ and $CD8^+$ $CD137^+$ $CD69^+$ activated T cells were evaluated within pre-expansion and expanded T cells following S1 stimulation in the four participants. Then the frequencies of $CD3^+$ $CD107^+$, $CD4^+$ $CD107^+$, and $CD8^+$ $CD107^+$ cytotoxic spike reactive T cells were measured within pre-expansion and expanded T cells following S1 stimulation in the four participants. Finally, the Frequencies of $CD4^+$ and $CD8^+$ naïve ($CCR7^+CD45RA^+$), central memory ($CCR7^+CD45RA^-$), effector memory $CCR7^-CD45RA^-$), and terminally differentiated memory ($CCR7^-CD45RA^+$) were evaluated within pre-expansion and expanded T cells following S1 stimulation in the four participants.
